# Preparing for the crewed Mars journey: microbiota dynamics in the confined Mars500 habitat during simulated Mars flight and landing

**DOI:** 10.1186/s40168-017-0345-8

**Published:** 2017-10-04

**Authors:** Petra Schwendner, Alexander Mahnert, Kaisa Koskinen, Christine Moissl-Eichinger, Simon Barczyk, Reinhard Wirth, Gabriele Berg, Petra Rettberg

**Affiliations:** 1 Radiation Biology Department, Institute of Aerospace Medicine, German Aerospace Center e.V. (DLR), Linder Höhe, 51147 Cologne, Germany; 2 Institute for Microbiology, University of Regensburg, Universitaetsstrasse 31, 93053 Regensburg, Germany; 3 Institute of Environmental Biotechnology, Graz University of Technology, Petersgasse 12/I, 8010 Graz, Austria; 4 Medical University of Graz, Department of Internal Medicine, Auenbruggerplatz 15, 8036 Graz, Austria; 5 BioTechMed-Graz, Mozartgasse 12/II, 8010 Graz, Austria; 6 Present address: UK Center for Astrobiology, University of Edinburgh, School of Physics and Astronomy, Peter Guthrie Tait Road, Edinburgh, EH9 3FD UK

**Keywords:** Mars500, Microbiota, Built environment, Mars flight simulation

## Abstract

**Background:**

The Mars500 project was conceived as the first full duration simulation of a crewed return flight to Mars. For 520 days, six crew members lived confined in a specifically designed spacecraft mock-up. The herein described “MIcrobial ecology of Confined Habitats and humAn health” (MICHA) experiment was implemented to acquire comprehensive microbiota data from this unique, confined manned habitat, to retrieve important information on the occurring microbiota dynamics, the microbial load and diversity in the air and on various surfaces.

In total, 360 samples from 20 (9 air, 11 surface) locations were taken at 18 time-points and processed by extensive cultivation, PhyloChip and next generation sequencing (NGS) of 16S rRNA gene amplicons.

**Results:**

Cultivation assays revealed a *Staphylococcus* and *Bacillus-*dominated microbial community on various surfaces, with an average microbial load that did not exceed the allowed limits for ISS in-flight requirements indicating adequate maintenance of the facility. Areas with high human activity were identified as hotspots for microbial accumulation. Despite substantial fluctuation with respect to microbial diversity and abundance throughout the experiment, the location within the facility and the confinement duration were identified as factors significantly shaping the microbial diversity and composition, with the crew representing the main source for microbial dispersal. Opportunistic pathogens, stress-tolerant or potentially mobile element-bearing microorganisms were predicted to be prevalent throughout the confinement, while the overall microbial diversity dropped significantly over time.

**Conclusions:**

Our findings clearly indicate that under confined conditions, the community structure remains a highly dynamic system which adapts to the prevailing habitat and micro-conditions. Since a sterile environment is not achievable, these dynamics need to be monitored to avoid spreading of highly resistant or potentially pathogenic microorganisms and a potentially harmful decrease of microbial diversity. If necessary, countermeasures are required, to maintain a healthy, diverse balance of beneficial, neutral and opportunistic pathogenic microorganisms. Our results serve as an important data collection for (i) future risk estimations of crewed space flight, (ii) an optimized design and planning of a spacecraft mission and (iii) for the selection of appropriate microbial monitoring approaches and potential countermeasures, to ensure a microbiologically safe space-flight environment.

**Electronic supplementary material:**

The online version of this article (10.1186/s40168-017-0345-8) contains supplementary material, which is available to authorized users.

## Background

Human exploration of our solar system started in 1957 with the launch of the first-ever satellite Sputnik by the Soviet Union. Another milestone was reached in 1961 when the first human, Yuri Gagarin, was sent to space [1]. Nowadays, after a number of robotic missions successfully having reached the Martian surface, a crewed Mars mission is considered one of the most important next steps for human space exploration.

The duration of a potential round-trip mission to Mars depends on the proximity of Earth and Mars to each other. One of the scenarios discussed is the opposition-class Mars mission, which can be carried out in approximately 520 days [2]. However, the opposition-class mission type allows only a short stay (approx. 30 days) on the Martian surface [3]. Once a mission will endure longer than 6 months and its target is beyond Earth orbit, it implicates new challenges for the safety of the crew, as well as the need for full autonomy, provision and reprocessing of resources. Besides numerous technical issues, one of the major challenges is the protection of the human crew from illness and infection caused by harmful biological contaminants.

Each human body is accompanied by 3.8 × 10^13^ microbial cells [4] and thus every crewed mission will include numerous microorganisms introduced by the humans’ “microbial cloud” [5]. This microbial cloud contains both microorganisms that are beneficial and can protect the human host from infection [6] but also harmful microorganisms posing several threats to crew’s safety:

Firstly, microorganisms and their biofilms might pose a risk for the integrity of materials and architecture [7, 8] by having the potential to destroy polymers and/or corrode metals directly or indirectly [9–12]. Once spacecraft components are damaged due to biocorrosion, adverse effects on avionics and spacecraft systems might result [13, 14]. Active biodegraders of various materials have already been found on-board the Mir [15]. Novikova [15] reported several cases of equipment failures on-board the International Space Station (ISS; e.g., deterioration of mechanic strength, alteration of dielectric or other properties) and identified common initiators of metal corrosion and polymer degraders that can make up 22.5 and 10% of the microbial community, respectively [16].

Secondly, spaceflight conditions, including confinement, stress and altered physical conditions such as microgravity, affect the human immune system [17] or can even cause an increased reactivation of latent viral infections [18, 19], potentially making the human crew more susceptible to infections.

Thirdly, confinement and the prevalent conditions during spaceflight might alter microbial growth and lead to undesirable accumulation and potential formation of biofilms on-board the space capsule [15, 20]. Various reactions and adaptations have been reported, including a shortened lag phase along with enhanced exponential growth [21–23], activation and aggregation of opportunistic pathogens [24], survival of prolonged desiccation [25], increased growth rate and/or elevated virulence [18, 26], development of antimicrobial resistance [27] or decreased susceptibility of microbes to antibiotics [28]. Uncontrolled microbial growth is a threat for space travel, as confirmed by reports on biofilm development in condensate behind panels on the Mir station [20], or reported fungal growth on-board the ISS at places where wet towels were hang to dry in close proximity to the wall [29]. Thus, potential bacterial infections of human tissues are considered a threat for the crew, as indicated from reports on infections of the urinary tract, upper respiratory tract and subcutaneous tissue occurring during human spaceflight on Mir or space shuttle [27]. Despite the fact that several microbiota monitoring experiments on the ISS have been launched (i.e. NASA’s “Microbial Observatory” project [30], JAXA’s “Microbe” experiment series [31] and ESA’s ARBEX/Extremophiles project [32]), there is still a lack of knowledge on how the microbiota responds to long-term confinement and how the structure and spreading changes when selective pressures occur [25].

It is assumed that confinement will particularly favour microbial transmission between crew members (the major microbial reservoirs) via surface contact and spreading through air [33, 34]. Additionally, in a confined and hygienically controlled environment (e.g. space station), the human-spread microbes will not face the same competition as in a natural open system populated by an established, strong and versatile environmental microbial community. These aspects might possibly favour the survival and spreading of microbial contaminants that may otherwise not survive. Moreover, micro-niches might harbour an accumulated microbial community, adapted to the specific environmental site with specific conditions, including surface material, humidity or concentration of nutrients.

As the ISS is not easy to reach and experiments cannot be performed in a straightforward set-up, current knowledge on confined microbial communities is sparse and thus risk estimations on crewed long-term spaceflight cannot be properly carried out [25]. A major step forward in assessing the risks and reducing them is simulation activities of such spaceflights on Earth, optimally accompanied by a comprehensive study of the microbial community and its dynamics.

A number of ground-based mock-up spacecraft and simulation habitats have been built mimicking most conditions prevalent during a spaceflight. Examples for such confined habitats are the Antarctic Concordia Station and isolation facilities like ILMAH, an inflated lunar/Mars analogue habitat, the HI-SEAS (Hawai’i Space Exploration Analog and Simulation) isolation habitat and the herein investigated Mars500 facility [35–37].

Similar to the ISS or the Mir station, these habitats function as closed systems, the confined crews experience unique stressors which could directly affect their health (resulting, e.g. in stress, fatigue, indisposition), their performance, and thus the fulfilment of tasks and the mission success. In contrary to open environments, confined habitats have restrictions on waste disposal, water and fresh air supply, as well as on personal hygiene. The unusual environmental conditions may result in bad air quality, water condensation or accumulation of biological residues and formation of microbial biofilms [35].

The Mars500 programme was developed as a multi-stage, ground-based simulation experiment of a return flight to Mars. It started with a 14-day isolation in 2007 to test the working capacity and reliability of operational procedures, the technical, medical and communication systems, and whether suitable space-flight simulation conditions for crew’s life were created during isolation.

The second stage was a 105-day confinement study of a crew consisting of six males in 2009. Its purpose was to acquire scientific and technical baseline information, while simulating all stages of a crewed flight to Mars. For the first time, microbiological and sanitary-hygiene studies were implemented to test technologies that allow rapid, cultivation-based diagnosis of the microbial community and its influence on the artificial habitat.

These preliminary test runs paved the way for the final simulation of the 520-day manned mission to Mars, which started on 3rd of June, 2010.

During the following 520 days, until 5th of November, 2011, the six crew members, also called marsonauts, followed a strict diet and schedule. Therein, they controlled the water processing units, the life support and air control system and carried out cleaning and maintenance tasks. To mimic the landing on the Martian surface, the crew was split into two groups of three people with one group entering the Martian simulation module (EU-50) from 1st to 27th of February, 2011. Furthermore, they actively performed scientific experiments in which they themselves were subjects for a number of psychological and physiological tests.

One of these experiments, which is described herein was the “MIcrobial ecology of Confined Habitats and humAn health” (MICHA) experiment, was designed to acquire detailed microbiota data from a confined manned habitat. In total, 360 samples from 20 (9 air, 11 surface) locations were taken at 18 time-points and processed by cultivation, PhyloChip and next generation sequencing (NGS) of 16S rRNA gene amplicons. We hypothesized that the microbial community will undergo severe changes during confinement, shaped by the extreme conditions in an unusual confined environment. Our study was conceptualized to serve as an important data collection for (i) future risk considerations in crewed space flight, (ii) an optimized design and planning of a spacecraft mission and (iii) the selection of appropriate microbial monitoring approaches and potential countermeasures in order to ensure a microbiologically safe space-flight environment.

## Methods

### Sampling location

**Fig. 1:**
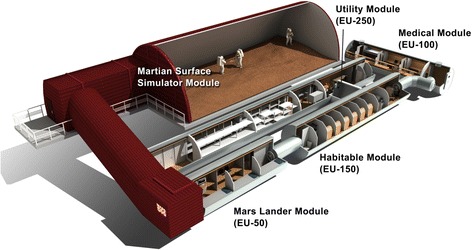
Illustration of the medical-technical facility (Mars500 Habitat) at the Institute for Biomedical Problems in Russia, Moscow, with its four experimental unit modules and the simulated Martian surface (SMS) module. © Adrian Mann/bisbos.com (approved)

Samples were taken during the first real-time (520 days) human isolation study mimicking a manned mission to Mars, called Mars500. The 520-day long experiment started on 3rd of June 2010 and was conducted at the medical-technical facility of the State Scientific Center of the Russian Federation—Institute for Biomedical Problems within the Russian Academy of Sciences (IBMP RAS) site in Moscow, Russia. During the isolation period, the crew, consisting of six male “marsonauts”, remained confined until 4th of November, 2011. The layout of the isolation facility, mimicking a spacecraft, was comprised of four hermetically sealed habitat modules and an additional simulated Martian surface module (see Fig. 1). The habitat modules (total volume 550 m^3^) were interconnected with each other, each of them equipped with its own life support system and serving as experimental units (EU). Besides the habitat modules, where the sampling was carried out (modules EU-100, EU-150 and EU-250), the facility also included an operation room, technical facilities and offices. The detailed description of each EU can be found in Additional file 1: Doc S1. Briefly, module EU-250 (referred to as utility module) contained a storage area and a gym, the habitable module EU-150 was comprised of the individual compartments, the community room and the kitchen, whereas in the medical module EU-100, medical and psychological experiments were conducted. The environmental parameters (i.e. O_2_ and CO_2_ concentration, relative humidity and temperature) of the four modules were regulated separately and controlled every week. However, as respective measurement points of microclimate variables were not coordinated with microbial sampling events an intensive data evaluation was omitted to prevent data over-interpretations. Briefly summarized, temperatures varied between 18.9 and 25.1 °C, relative humidity from 35.2 to 53.8%, CO_2_ and O_2_ pressure were in a range of 0.05–0.53% and 20.5–20.9%. The modules varied only slightly from each other.

### Sampling

**Fig. 2:**
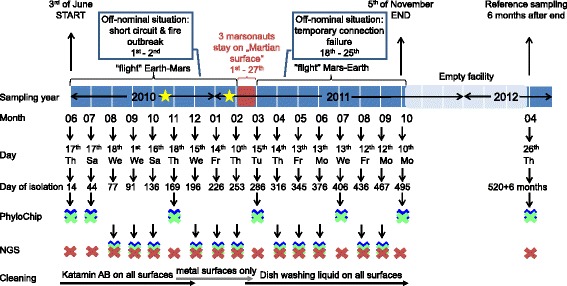
Timeline of the Mars500 experiment from the beginning (3rd of June, 2010) until the end (5th of November, 2011). The schematic drawing also indicates important steps and events during the confinement (above timeline) including the two off-nominal situations (critical situation simulations) and sampling dates from 18 sampling events. Red area/font denotes the stay of three marsonauts in the simulated Martian surface complex, whereas light blue area represents the timeframe where the facility was untenanted. One reference sampling was performed 6 months after confinement. Crosses represent samples that were used for PhyloChip analyses or NGS, respectively. Samples from each sampling were subjected to cultivation experiments. Red: medical module EU-100; green: habitable module EU-150; blue: utility module EU-250. Yellow stars indicate changing of NANO-filters and cleaning events of the primary filters on day 162 (11th of November, 2010) and 243 (2nd of February, 2011) of isolation

In order to determine the microbial load and biodiversity in the air and on surfaces, as well as their changes over time, air and surface samples were collected by marsonaut Charles Romain on a monthly basis. The sampling period during the isolation experiment started on the 17th of June, 2010 (isolation day 14), and ended on the 10th of October, 2011 (isolation day 495). An additional reference sampling was performed 6 months post-confinement on 26th of April, 2012 (see Fig. 2). Nine sites, chosen from three of the four habitat modules (EU-100, EU-150 and EU-250), were surveyed during this study to compile an overview of the bacterial airborne contamination present in the Mars500 facility, whereas eleven areas were selected for monitoring the natural colonization of surfaces (Table 1, and for photographs of the sampling locations, see Additional file 2: Figure S1).

**Table 1:** List of air and surface samples and description of the corresponding sampling area at the Mars500 facility (EU-250 = utility module, EU-150 = habitable module, and EU-100 = medical module)

Module	Air sample no.	Description	Surface Sample no.	Description	Surface character	Orientation
250	1	On the floor in front of the toilet	2	Wall above the vanity basin	Stainless steel	Vertical
	6	On the floor of the gym next to the treadmill	3	Wall under the faucet close to the corner in the per capita shower cabin	Stainless steel	Vertical
	7	On the floor in front of the greenhouse	9	Left hand side of the inside of the greenhouse	Stainless steel	Vertical
	8	On the floor between storage racks	11	Rack surface from the storage area of clothes	Stainless steel	Horizontal
	9	On the floor between the fridges				
150	3	On the carpeted part of the floor in the community room	1	External surface of the toilet bowl	Stainless steel	Horizontal
	4	On top of the table in the dining area	4	Wall in the corner in the community cabin	Wood	Vertical
	5	On the floor of an individual compartment	5	Desktop surface close to the keyboard on the left hand side in the main panel	Wood	Horizontal
			6	Surface of the dining table in the kitchen	Wood	Horizontal
			10	Table surface in individual compartment	Wood	Horizontal
100	2	On the floor at the working place between desk and bunk	7	Table surface close to the insulator zone	Wood	Horizontal
			8	Table surface around water plum	Wood	Horizontal

### Air sampling

Samples were taken using the active air sampler Sartorius AirPort MD8 (Sartorius AG, Goettingen, Germany) and gelatine air filters (17528-80-ACD, Sartorius AG, Goettingen, Germany). At each sampling site, 500 l air were filtered with a flow rate of 30 l per minute.

### Surface sampling

Surface sampling with swabs was performed according the ECSS-Q-ST-70-55C standard [38] applied in spacecraft-associated cleanrooms for assessment of the mesophilic aerobic microbial load.

The swab (552C regular swab; ethylene oxide sterilized, Copan, Brescia, Italy) was moistened with PCR grade H_2_O. An area of 5 × 5 cm^2^ was sampled in three directions (horizontal, vertical and diagonal). During this procedure, the swab was turned several times. For downstream cultivation analysis, the swab was broken at the predetermined breaking point and was transferred to a sterile 15-ml Falcon tube (VWR International GmbH, Darmstadt, Germany) containing 2.5 ml PBS (wet swab). For downstream molecular analysis, a second swab sample (dry swab) was taken adjacent to the area swabbed for cultivation. The swab was placed back in its original, still sterile container.

### Controls

Appropriate field controls were taken by waving the sampling tool (air filter or swab) through the air at the Mars500 facility for a few seconds, representing so-called field blanks. This procedure was performed at least once per sampling event. Unused sampling material was processed along with the samples and served as lab controls.

### Sample storage until processing

Upon completion of the air and surface sampling, all samples (gelatine filters, wet and dry swabs) from one sampling event were placed into a hatch within the isolation facility, allowing access to the samples from the outside. After closing the hatch from inside, samples were removed the same day and stored at − 80 °C until processing.

### Sample processing for downstream cultivation approach

#### Air samples

The applied assay for assessment of vegetative mesophilic aerobic microbial load was performed according to the ECSS-Q-ST-70-55C [38] standard. After gentle thawing of the samples, the gelatine filter was aseptically placed onto the surface of R2A plates. Incubation was carried out for 72 h at 32 °C (± 1 °C). Colony counts were taken every 24 h, performing the final count after 72 h.

#### Surface samples

After gentle thawing, each sample vial containing 2.5 ml PBS and a swab was vortexed at maximum power for 5 to 6 s and the liquid was divided into two aliquots (1 and 1.5 ml). One millilitre was used to determine the overall microbial cultivables, whereas 1 ml thereof was subjected to heat-shock (HS) treatment. In order to determine the overall microbial “vegetatives”, two aliquots of 0.5 ml each were aseptically pipetted onto the surface of two R2A petri plates. Colony-forming unit (CFU) counts were performed after incubation at 32 °C (± 1 °C) for 24 and 48 h, and the final count was done after 72 h. A heat-shock step was included to retrieve the fraction that survives the heat-shock treatment, following the considerations of the NASA and ESA guidelines for planetary protection-relevant contamination measurements. Therefore, the remaining sample was placed in a water bath at 80 ± 2 °C for 15 min. Following heat-shock treatment, the sample was cooled rapidly to 30–35 °C, vortexed again at maximum power for 2 s and further steps followed as described above for “vegetatives”.

#### Processing of isolates

After the final counting of CFUs, up to three morphologically different colonies (size, shape, texture, colour, raised, concave etc.) were picked from each plate to cover the broadest diversity. Bacterial specimens were isolated and purified from mixed environmental cultures using the streak plate method. Once purified, the strains were sent to LGC Genomics (Berlin, Germany) for taxonomic classification based on traditional Sanger sequencing of almost full-length 16S rRNA gene. The 16S rRNA gene was amplified with the primer set 27F (5′-AGRGTTTGATCMTGGCTCAG-3′, [39]) and 1492uR (5′-GGWTACCTTGTTACG ACT T-3′, [39]).

Sequences retrieved from microbial isolates were trimmed (min length 700 bp) and classified against GreenGenes database (for comparison with PhyloChip data, updated version [40]) or SILVA (version 128, for comparison with NGS data [41, 42]). One sequence per identified species served as the representative strain. All sequences were submitted to Genbank and are publicly available (accession numbers KF777358 to KF777686, and KJ187479 to KJ187482).

#### Sample processing for DNA extraction and PhyloChip analysis

Genomic DNA was extracted from the swabs and sent to Second Genome, Inc. (South San Francisco, CA, USA) to perform PhyloChip analysis. To maximize the genomic DNA (gDNA) yield and receive reliable results, dry swabs taken per module per sample event were pooled. DNA extraction was performed according the protocol established by Tillet and Neilan [43] and optimized by Stieglmeier et al. [44]. DNA samples for DNA microarray were processed as described briefly below: gDNA concentration was determined using PicoGreen® method. Bacterial 16S rRNA genes were amplified in duplicate using Molzym™ 16S Basic Master Mix (Molzym GmbH & Co. KG, Bremen, Germany). Amplicons were concentrated via the solid-phase reversible immobilization method and purified using PowerClean® DNA Clean-Up Kit (MO BIO Laboratories, Inc., Carlsbad, CA, USA). PCR amplification products were quantified by electrophoresis using the Agilent Bioanalyzer® (Agilent Technologies, Inc., Santa Clara, CA, USA). PhyloChip™ Control Mix was added to each sample. Bacterial amplicons were fragmented, biotin labelled and hybridized to the PhyloChip™ Array version G3. Arrays were washed, stained and scanned using a GeneArray® scanner (Affymetrix, Santa Clara, CA, USA). Affymetrix software (GeneChip® Microarray Analysis Suite) was used to measure hybridization values and fluorescence intensities. Please refer to Hazen et al. [45] supplementary methods for a full description of the PhyloChip design.

#### PhyloChip data analysis

After rank normalization of fluorescence intensities across probes for each individual array, data were pre-processed according to DeSantis et al. [46] and Hazen et al. [45], i.e. filtering for taxa that are present in at least one sample or for taxa that show significant abundance differences. The false discovery rates were determined by calculating *q* values using Benjamini-Hochberg procedure [47]. Operational taxonomic unit (OTU) determination was based on the novel empirical OTU (eOTU) selection process, i.e. directly taxonomically annotated with a Bayesian method from the combination of the 9-mers contained in all probes of the set [48]. Therefore, probe sets were defined on the basis of relatedness of the probes and their correlation in fluorescence intensity throughout the experiment. For further analysis, either abundance metrics or binary metrics were generated (for detailed information please refer to Hazen et al. [45] supplements). Inter-sample distances are based on Bray-Curtis. The Second Genome’s PhyCA-Stats™ analysis software package was used to perform multivariate data analysis. The graphical processing of the dissimilarity scores was done by generating hierarchical clustering maps using the average neighbour (HC-AN) method and non-metric multidimensional scaling (NMDS). Unless stated otherwise, significance testing was performed using the Adonis test.

Besides the general analysis, a correlation of OTU trajectories with metadata was carried out. Metadata included information on sampling date and CFU data obtained from cultivation. A selection of eOTUs which had a significant correlation with different metadata factors was done by Spearman rank correlation.

#### Sample processing for DNA extraction and next generation sequencing analysis

Swab samples not used for PhyloChip analysis were subjected to NGS via Illumina HiSeq amplicon sequencing. Genomic DNA from 146 samples, including 10 field blank control samples, was extracted using FastDNA SPIN Kit (MP Biomedicals, USA) according to manufacturer’s instructions. Additionally, we processed two DNA extraction kit controls to assess the contamination level introduced by the materials (“kitome”). The concentration of isolated DNA was quantified with Qubit dsDNA HS Assay Kit (Thermo Fisher Scientific, USA).

#### 16S rRNA gene amplicons for NGS

Extracted DNA was amplified in a first PCR with the primer pair 515f (GTGYCAGCMGCCGCGGTAA) and 926r (CCGYCAATTYMTTTRAGTTT) targeting the complete V4 region of the 16S rRNA gene [49, 50]. Each forward and reverse primer contained a specific primer pad (TATGGTAATT/AGTCAGCCAG) and linker (GT/GG), as described in the protocols and standards section of the Earth microbiome project [49]. PCR reactions (30 μl) were executed in triplicate and comprised 22.4 μl PCR grade water, 6 μl Taq&Go™ Mastermix (MP Biomedicals, Heidelberg, Germany), 0.3 μl of forward and reverse primers each (10 μM) and 1 μl extracted DNA template (0.1–1.6 ng/μl). Amplifications were conducted in 35 cycles on a Whatman Biometra® Tpersonal and Tgradient thermocycler (Biometra GmbH, Göttingen, Germany) and a TECHNE TC-PLUS gradient thermocycler (Bibby Scientific Ltd., Stone, UK) with the following settings: 95 °C for 45 s, 55 °C 45 s, 72 °C 90 s, including an initial denaturation of 3 min at 95 °C and a final extension of 5 min at 72 °C. PCR products of respective samples and controls were pooled, and the quality was checked by gel electrophoresis. If the quality (amount, concentration) of the PCR product obtained from an individual swab sample was found to be insufficient, it was combined with all other samples within a module of a respective sampling time-point to cover the timeframe of isolation as continuously as possible. Hence, the NGS analysis covered individual as well as pooled swab samples, where the latter served as a baseline regarding influences of different sampling locations, materials and positions on the overall structure of the microbiota.

For multiplexing, sample specific Golay barcodes were attached to the specific primer pad on forward and reverse primers respectively in a second PCR. Three microlitres of the first PCR products (pooled) was amplified in 15 cycles and four replications of 50 μl with the following cycling conditions: 95, 53 and 72 °C for 30 s respectively. Settings for initial denaturation and final extension are given above as well as the composition of the reaction mix (30 μl). After quality checking the final PCR products by gel electrophoresis, all four independent reactions per sample were pooled and purified according to the protocol of the Wizard SV Gel and PCR Clean-Up System (Promega, Madison, USA). Equimolar DNA concentrations of each barcoded amplicon were sent to GATC Biotech AG, Konstanz, Germany. After entry quality control and adapter ligation, 16S rRNA gene amplicons were sequenced on an Illumina HiSeq instrument using an optimized protocol to achieve 300 bp paired end reads in the rapid run mode. Sequences were sorted by the company according to inline barcodes, joined and stitched.

#### Diversity analysis of 16S rRNA gene amplicons

Stitched sequences were analysed with QIIME 2 (2017.4 release) and QIIME 1.9.1. [51] according to tutorials provided by the QIIME developers. After checking read quality with fastqc, barcodes were extracted and reads as well as metadata were imported to QIIME 2. The DADA2 algorithm [52] was used to demultiplex, denoise truncated reads (400 bp length, including phiX and chimera filtering), and to generate ribosomal sequence variants (RSVs), which were then summarized in a feature table. This procedure allows for a higher resolution and more accurate estimates of diversity and composition than common methods using clustering steps to generate OTUs at a certain similarity percentage. Feature tables were rarefied to a depth of 1000 RSVs before controls were manually subtracted from respective sample groups (time-points) and feature tables. Filtered rarefied feature tables served as input for following alpha and beta diversity analysis and statistics using the QIIME 2 core diversity metrics. For phylogenetic metrics, representative sequences were aligned with the mafft program and a phylogenetic tree was generated with FastTree after the multiple sequence alignment was masked and filtered. The taxonomic analysis was based on a customized naïve-bayes classifier trained on 16S and 18S rRNA gene OTUs clustered at 97% similarities within the Silva123 database release and trimmed to a length of 400 bp to fit to the cut-off used for denoising in DADA2 (see above). Differential abundances of taxa were identified by analysis of composition of microbiomes (ANCOM [53]). Statistics were calculated through QIIME2 (Kruskal-Wallis tests, PERMANOVA tests, Spearman rank correlations), and supported with calculations in QIIME 1.9.1. (MRPP, Adonis, ANOSIM), and R (BioEnv – BEST [54]) using 999 permutations were applicable. Microbial phenotypes were predicted with BugBase [55], a software that relies on the tools PICRUSt, IMG, KEGG and PATRIC.

## Results

Microbial monitoring of crewed spacecraft and spacecraft-related confined habitats is essential to maintain a safe, non-hazardous environment for the crew [56]. Until now, little is known about the influence of long-term confinement on the microbial inhabitants and their community structure and whether the structure of the microbiota undergoes changes with time. Thus, obtaining information about resident microbial diversity is critical in order to:

1. Advance our understanding of the overall microbiota present in a crewed habitat,
2. Obtain detailed information of the community structure and its economical dynamics,
3. Identify the sources of microbial contamination and microbial transmission between the modules,
4. Determine whether the confined habitat met hygienic standards.

In addition it may help us to:

5. Evaluate potential jeopardy caused by harmful microorganisms.

Mars500, the long-term ground simulation experiment of a crewed flight to Mars, provided a unique opportunity to acquire microbiota data from a completely sealed manned habitat over 520 days. The inhabiting microbial community was assessed by cultivation and molecular state-of-the-art techniques such as PhyloChip G3 and next generation sequencing.

### Cultivation reveals a fluctuating microbial load

The use of a standardized sampling and cultivation procedure for all sampling sites allowed tracking of changes over the whole time, and the quantitative and qualitative comparison of the microbial load of all sampling sites and modules. In particular, concerning the spread of microorganisms and their further development in a closed crewed habitat, it is important to pinpoint hotspots of microbial accumulation. Air and surface samples taken from the habitable (EU-150), utility (EU-250) and medical (EU-100) modules throughout the confinement were analyzed with respect to their cultivable microbial load (Table 1, Additional file 2: Figure S1). Field blank samples served as a control and analyses thereof demonstrated a sterile handling of the gelatine filters and swabs during sampling procedure.

*Surface contamination* was monitored once a month during the confinement with one additional reference sampling after the confinement period (April 2012). Colony-forming units (CFUs) from 0 to 2.9 × 10^4^ per 10 cm^2^ were observed with a mean value for all samples of 6.7 × 10^2^ CFUs per 10 cm^2^ (*n* = 198).

**Fig. 3:**
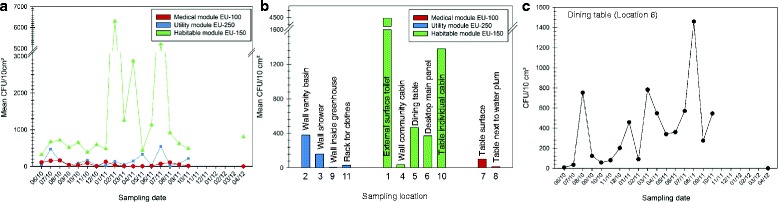
CFUs per 10 cm^2^ surface, appearing on R2A after 72 h incubation at 32 °C. **a** Mean CFU values (*y*-axis), whereas different sampling locations within one module were grouped for each sampling event (*x*-axis). **b** The mean CFU values (*y*-axis) of all sampling events for each sampling location (*x*-axis). **c** The CFU values (*y*-axis) from a representative sample location (dining table, location 6) for each sampling event (*x*-axis)

The highest number CFUs over time were observed in the habitable module (EU-150) with counts up to 1.1 × 10^4^ CFUs per 10 cm^2^. Counts retrieved from the other modules were consistently 23- to ninefold lower, with max. 1.9 × 10^3^ CFUs in the utility module (EU-250) and 3.2 × 10^2^ CFUs in the medical module (EU-100) per 10 cm^2^, respectively (Fig. 3).

However, the microbial cultivable load was subject to severe fluctuations with respect to time-point of sampling, module and detailed location therein (see Fig. 3). Mean CFU values of each individual sampling location considered over time (18 time-points) ranged between 36 and 4.472 per 10 cm^2^ (habitable module EU-150), 5 to 3.8 × 10^2^ CFU per 10 cm^2^ (utility module EU-250) and max. 1.0 × 10^2^ per 10 cm^2^ (medical module EU-100), respectively (Fig. 3b).

The accumulation in the habitable module (EU-150) was up to 100 times higher in samples from the toilet (location 1) compared to the table in community room (location 4). The highest mean CFU counts were retrieved from toilet (location 1), desktop (location 5) and the individual compartment (location 10; all from the habitable module EU-150; Additional file 3: Table S1).

The microbial load on the surfaces revealed three individual peaks of high contamination in module EU-150, in particular right before and after the simulated Mars landing (peak 02/11, 04/11), followed by an extremely low overall CFU count and an increase in 07/11. The last peak is also accompanied by an increase in CFUs within the utility module.

An overall mean reduction of 85% was observed on CFU number when samples were subjected to a heat-shock treatment at 80 °C (15 min). Notably, only 2% of the total microbial load survived the heat-shock from samples taken at the toilet (location 1), the dining table (location 6) and the table in the individual compartment (location 10), whereas almost all cultivable microorganisms from the greenhouse (location 9) grew after the incubation at 80 °C, indicating the potential higher abundance of spore-forming microorganisms therein.

**Fig. 4:**
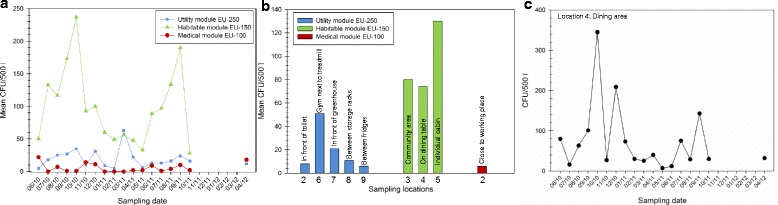
CFUs retrieved from 500 l air, appearing on R2A after 72 h incubation at 32 °C. **a** Mean CFU values (*y*-axis), whereas different sampling locations within one module were grouped for each sampling event (*x*-axis). **b** The mean CFU values (*y*-axis) of all sampling events for each sampling location (*x*-axis). **c** The CFU values (*y*-axis) from a representative sample location (dining area, location 4) for each sampling event (*x*-axis)

*Air contamination* was monitored simultaneously with surface sampling. Nine areas were surveyed in the three different modules (Fig. 4; Additional file 4: Table S2). Air samples revealed cell numbers from 0 to 7.2 × 10^2^ per m^3^ with an average value of 86 CFU per m^3^ (*n* = 162). Consistent with the data from the surface samples, the highest abundance of airborne contaminants was detected in the habitable module (EU-150; 14 to 7.2 × 10^2^ CFU per m^3^). A far lower microbial burden was obtained from the medical module (EU-100; 0 to 44 CFU per m^3^) and the utility module (EU-250; 0 to 5.4 × 10^2^ CFU per m^3^; Fig. 4a). Similar to the surface samples, the amount of CFUs retrieved from air was also subject to fluctuations with respect to location and time-point. However, the peaks observed in surface samplings (Fig. 3) did not correlate with peaks observed in air.

Looking at the airborne fraction of each module, the highest number of cultivable bacteria was obtained from samples in the community room (location 3), dining area (location 4) and individual compartment (location 5, Fig. 4b). These locations represent areas with a high nutrient content (food debris) and are characterized by a high dispersion of dust caused by human activity, showing a sixfold and a 53-fold mean increase compared to the utility module (EU-250) and medical module (EU-150), respectively. All samples from the utility module revealed comparatively low CFU counts apart from sample 6 where the air sampler was placed on the floor of the gym close to the treadmill (Fig. 4b).

### Staphylococci dominated the airborne cultivable diversity, bacilli and staphylococci dominated the surfaces

A full overview of all retrieved isolates, the location and time-point of respective sampling is given in Additional file 5: Table S3.

**Fig. 5:**
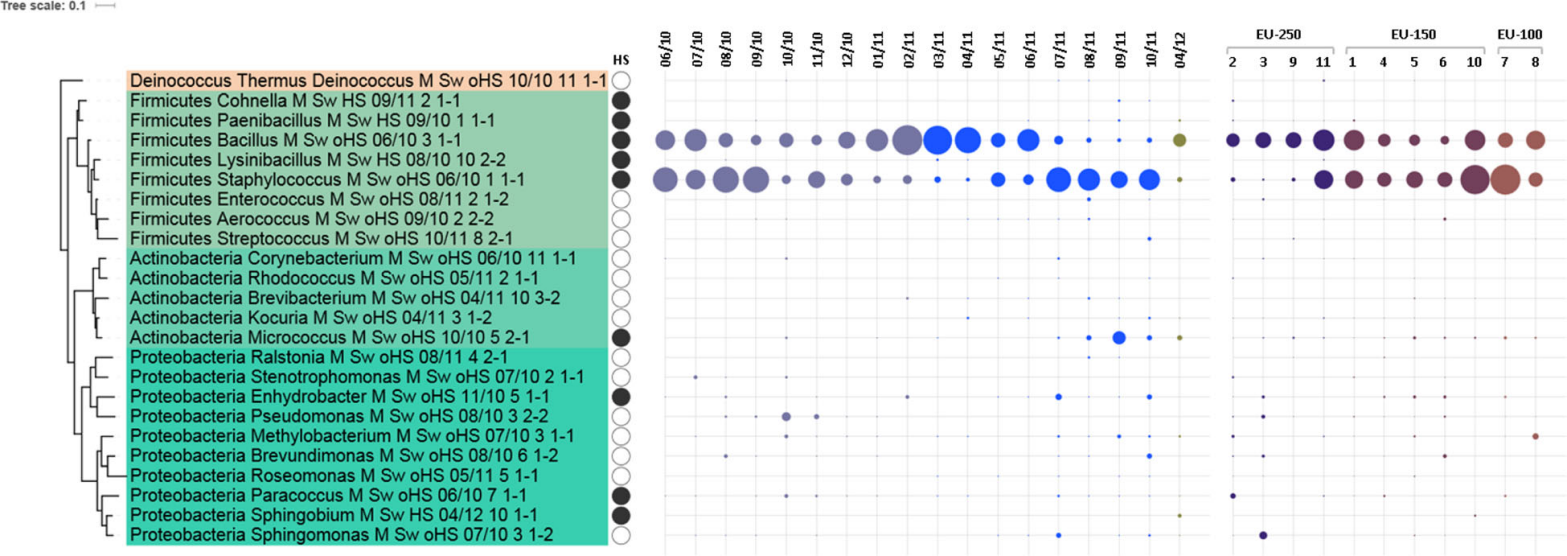
Isolates from surfaces, only those that appeared at least with three CFUs; filled circles next to the isolate names indicate survival of heat shock (representatives of this genus were found to survive this treatment). The number of isolates retrieved is visualized by the size of the dots; respective appearance was ordered according to time point of sampling (different colours reflect time before landing and after; reference sampling in 04/12) and location. Figure was prepared via iTol [126]

After quality checking, 443 isolate sequences were analyzed and assigned to the five phyla Actinobacteria, Bacteroidetes, Firmicutes, Proteobacteria and Deinococcus-Thermus (Additional file 5: Table S3). All were represented in the habitable EU-150 and utility EU-250 module, whereas only three phyla (Bacteroidetes, Proteobacteria and Deinococcus-Thermus) were detected in the medical module EU-100. Thirty-six different genera were detected, indicating a high overall diversity covered by only one enrichment condition. An overview of the microbial genera that appeared at least three times is given in Fig. 5. This figure also displays the distribution over time and location.

Ten of the detected microbial genera, namely *Aerococcus, Bacillus, Corynebacterium, Enhydrobacter, Methylobacterium, Microbacterium, Micrococcus, Paracoccus, Pseudomonas* and *Staphylococcus*, were enriched from all three modules, with *Bacillus* and *Staphylococcus* being most abundant. Notably, these two genera revealed an antagonistic pattern when regarded over time (Fig. 5).

At the species level, 47 different taxa were identified, with a core microbiota being present in all modules: *Bacillus amyloliquefaciens*, *Bacillus safensis*, *Micrococcus luteus*, *Paracoccus yeei*, *Pseudomonas libanensis*, and the *Staphylococcus* species *S. aureus*, *S. cohnii*, *S. epidermidis*, *S. haemolyticus* and *S. hominis*. Nevertheless, each module revealed a specific bacterial signature as well.

The highest microbial diversity was observed in the utility module (EU-250; 34 species), followed by the habitable module (EU-150; 30 species), whereas only 15 species were detected in the medical module (EU-100).

The vast majority of the identified isolates of all three modules were Gram-positives, whereas less than one quarter (approximately 20%) were Gram-negative bacteria. This trend of distribution was nearly identical for all three modules.

*Bacillus* species were particularly resistant to the applied heat-shock, as also indicated in Fig. 5, but also non-spore forming microorganisms, such as *Micrococcus*, *Enhydrobacter*, *Paracoccus* etc. were found to survive this procedure. In all three modules, the spore-forming strains accounted for approximately 70%.

Compared to surface diversity, the airborne isolates were less diverse. In total, three different phyla from 274 airborne isolates were detected encompassing only 15 genera. Ninety percent of the isolates from the habitable and utility module were representatives of the Gram-positive phyla. The majority of the cultivated bacteria (91% of the isolates, with *Staphylococcus* predominating) belonged to the Firmicutes. Proteobacteria (only α- and γ-Proteobacteria) constituted 6% of the cultivable species, whereas Actinobacteria representatives accounted for 3%. On the genus level, only staphylococci *(S. aureus*, *S. epidermidis*, *S. haemolyticus*, *S. hominis*) were detected in all three modules. Staphylococci accounted for the majority (95.5%) of all processed sequences in the habitable module (EU-150), whereas the modules that contained a lot of equipment and consisted of areas for storage revealed a lower content (66 and 62% for utility EU-250 and medical module EU-100, respectively). In all, the medical module revealed the lowest microbial airborne diversity, whereas the microbial communities of the habitable and utility modules were more manifold.

*Staphylococcus* representatives were the most abundant isolates retrieved. Although they were clearly dominating the airborne microbial diversity throughout the confinement, surfaces were shared with mainly *Bacillus* species, with time-dependent dynamics observed. As airborne microorganisms are mostly associated with particles [57], we can propose an increased distribution of staphylococci through the air by skin flakes.

### PhyloChip G3 analysis revealed a time- and location-dependent, fluctuating proteobacteria-dominated microbial community

For monitoring purposes based on molecular information, we selected seven sampling events, namely days 14 (04/10), 44 (07/10), 169 (11/10), 286 (03/11), 406 (07/11), 495 (10/11) and 520 + 6 months (04/12), for PhyloChip G3 analysis (see Fig. 2).

Each PhyloChip sample contained pooled surface samples from one module taken at a certain sampling event, i.e. five swabs from the habitable (EU-150) or four swabs from the utility (EU-250) module, respectively. Samples from the medical module (EU-100) were not included.

A total of 1196 empirical operational taxonomic units (eOTUs) were retrieved (for a complete list see Additional file 6: Table S4). The HybScore for an eOTU was calculated as mean fluorescence intensity of the perfectly matching probes, exclusive of the maximum and minimum [48]. Bray-Curtis based non-metric multidimensional scaling NMDS was performed to identify the negative control as outlier sample (stress: 0.1298 abundance). The small number of taxa (71) detected therein pointed towards an adequate sterile handling during sampling and DNA extraction; these taxa were subtracted from subsequent data analysis.

Ninety-five percent of the remaining 1125 eOTUS were assigned to four phyla: Proteobacteria (41%, mainly γ-Proteobacteria, followed by α- and β-Proteobacteria), Firmicutes (34%, two thirds represent *Clostridia*, remaining were bacilli and unclassified taxa), Bacteroidetes (11%, mainly *Prevotella*) and Actinobacteria (8%, almost exclusively *Corynebacteria*).

The distribution of those four phyla was similar for both modules, but significant differences between the modules were revealed at more resolved taxonomic levels. A significantly greater diversity in bacterial genus richness was detected in the utility module EU-250 (non-paired, heteroscedastic Student’s *t* test, *p* value < 0.05) compared to the habitable module EU-150. For example, the bacterial genus richness for samples from the habitable module EU-150 ranged from 101 to 139 and from 130 to 171 in the utility module EU-250.

For both modules, fluctuations in the microbial community structure were detected over time without showing a trend (Additional file 7: Figure S2). Contrary to results from NGS analyses (see below), these results did not support the hypothesis implying an increase or decrease of microbial diversity over time.

**Fig. 6:**
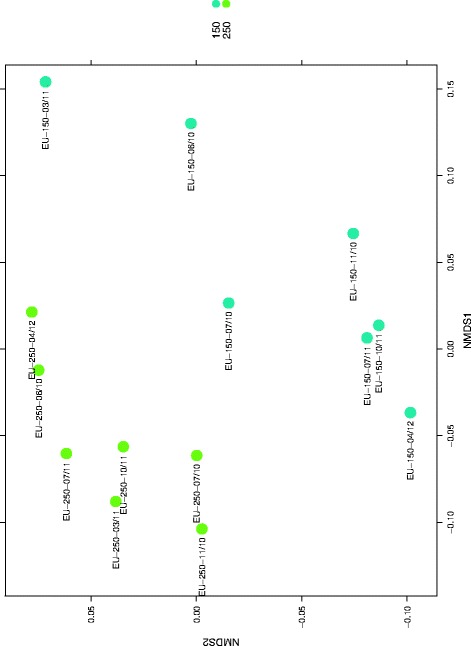
NMDS based on Bray-Curtis distance between samples based on the abundance of 1125 eOTUs present in at least one sample, stress = 0.1417

Βeta diversity analysis using a Bray-Curtis based NMDS approach on the abundance dataset revealed a clear separation of the microbiota of samples from the habitable module EU-150 versus the utility module EU-250 (stress = 0.1417, Adonis test, *p* = 0.003, Fig. 6).

To compare the two modules’ microbiota with each other, eOTUS were filtered to identify eOTUs that were significantly different (parametric Welch test: *p* value < 0.05) in one of the modules from the overall microbiota. 279 taxa passed the filtering and were used directly for abundance metrics.

**Fig. 7:**
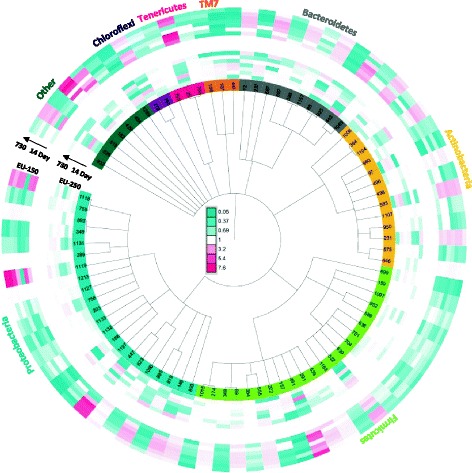
Interactive Tree Of Life (iTOL) based on 16S rRNA genes of 82 eOTUs that are significantly different (*p* values < 0.05) when comparing module EU-250 samples (inner rings) and module EU-150 samples (outer rings) [126]. The colour saturation indicates the degree of difference from the mean EU-250 value. Each layer of the two rings indicates a sampling time-point, with the earliest samplings closer to the centre of the tree

To visualize the differences on a phylogenetic basis, the iTOL tool was used (Fig. 7). The resulting 279 eOTUs were assigned to 69 bacterial families. One eOTU from each family was representatively selected that revealed the greatest difference between the two modules. However, within 13 families, eOTUs were detected that showed both significant increases and decreases in their relative abundances. Regarding these families, both eOTUs were picked as representatives (82 in total).

Exclusively, all eOTUs assigned to the candidate division TM7 group and Cyanobacteria (eOTU 932), Fusobacteria (eOTU 519), WS3 (eOTU 434) and OP11 (eOTU 1269) generally revealed a decrease in the habitable module EU-150. From the following phyla, only one representative was significantly higher abundant in the habitable module EU-150: Verrucomicrobia (eOTU 528), Planctomycetes (eOTU 182) and Synergistetes (eOTU 429).

The selected eOTUs that are representatives of the phyla Chloroflexi (50%), Tenericutes (50%), Bacteroidetes (50%), Actinobacteria (38%), Firmicutes (32%) and Proteobacteria (32%) exhibited mixed responses (numbers in brackets gives the percentage of eOTUS that are significantly increased in the habitable module EU-150).

Specifically, 26 eOTUs were significantly increased in the habitable module EU-150 and were identified as SHD-231 (eOTU number 272), *Clostridium sp.* (eOTU 25, 491), *Prevotella sp.* (eOTU 85), *Bacteroides vulgatus* (eOTU 442), *Bifidobacterium sp.* (eOTU 1006), *Actinomadura nitritigenes* (eOTU 496), *Dermabacter hominis* (eOTU 583), *Mobiluncus curtisii* (eOTU 231), *Leuconostoc fallax* (eOTU 522), *Peptoniphilus asaccharolyticus* (eOTU 539), *Dialister sp.* (eOTU 555), *Bacteroides ureolyticus* (eOTU 148) and *Brucella sp.* (eOTU 447). Twelve eOTUs remained unclassified on genus level.

The following eOTUs were significantly higher in abundance in the utility module EU-250: *Prochlorococcus sp.* (eOTU 932), *Luteolibacter sp*. (eOTU 528), *Planctomyces sp.* (eOTU 182), *Jonquetella anthropi* (eOTU 429), *Clostridium sp.* (eOTU 766), *Prevotella sp.* (eOTU 925), *Bacteroides sp*. (eOTU 1063), *Propionibacterium acnes* (eOTU 960), *Rothia dentocariosa* (eOTU 498), *Actinomyces hyovaginalis* (eOTU 950), *Corynebacterium sp.* (eOTU 646), *Gemella sp.* (eOTU 159), *Staphylococcus aureus* (eOTU 952), *Bacillus sp.* (eOTU 589), *Streptococcus sp.* (eOTU 701), *Lactobacillus sp*. (eOTU 704), *Granulicatella sp.* (eOTU 639), *Eubacterium sp.* (eOTU 157), *Peptostreptococcus sp*. (eOTU 222), *Novosphingobium sp. (*eOTU 1080), *Neisseria sp.* (eOTU 1197), *Polynucleobacter sp.* (eOTU 168), *Marinobacter sp.* (eOTU 756), *Pseudomonas sp.* (eOTU 1213, 289) and 30 remained unclassified on genus level.

Apart from location-specific patterns, Spearman rank correlations were performed to identify those eOTUs (out of 1125) that show a significant correlation with time in each module (for heatmaps see Additional file 8: Figure S3). In both modules, only a small fraction of eOTUs, i.e. a total of 57 in the habitable (EU-150) and 38 in the utility module (EU-250), revealed a significant time correlation.

While in the habitable module (EU-150), 25 eOTUs decreased over time and 32 eOTUs increased with proceeding confinement (see Additional file 8: Figure S3A). All eOTUs assigned to α-, β-Proteobacteria and Sphingobacteria (only Chitinophagaceae) strikingly abated during the confinement, whereas Actinobacteria (exclusively Corynebacteriaceae) and Clostridia (including mainly Lachnospiraceae) revealed an accumulation with time. Bacilli and Bacteroidia displayed mixed responses.

In the utility module (EU-250), 21 eOTUs revealed a negative correlation (Additional file 8: Figure S3B). A decline with increasing confinement duration was observed in 21 eOTUs that belonged to Firmicutes (13), Proteobacteria (4), Actinobacteria (3) and Bacteroidetes (1). However, after the facility was left unoccupied for 6 months, all of these eOTUs increased again and revealed greater HybScores in the post-confinement sampling. For 17 eOTUs, a reverse trend was detected. Those were less abundant in the beginning, showed a peak between 6 to 12 months during the isolation, and a decrease in samples from the post-confinement sampling in April of 2012. This eOTU group consisted mainly of human-associated Proteobacteria (12) and Firmicutes (5; Clostridia, *Enterococcus*).

In summary, the identified eOTUs mainly belonged to Firmicutes, Proteobacteria, Actinobacteria and Bacteroidetes and revealed a reverse trend in both modules. In contrast, the majority of representatives of the abovementioned taxa were increased in the habitable module (EU-150), decreased in the utility module (EU-250).

### Next generation sequencing revealed the presence of 402 microbial genera in the Mars500 modules and a dominance of *Corynebacterium*, *Ralstonia* and *Staphylococcus*

16S rRNA gene amplicon analysis of 118 samples (a total of 81 individual swab samples and 37 pooled samples) not only allowed a detailed survey of a changing microbiotae throughout the different modules but also sample resolution was increased, allowing for tracking of microbial patterns for individual locations, different material and orientation of the samples surface.

After quality filtering, the dataset comprised 1.2 million sequences (mean frequency = 10,149 sequences) and 1810 features (mean frequency = 662 features; see Additional file 9: Table S for more details on read statistics, as well as statistics on alpha and beta diversity). Overall, 402 features could be resolved to or beyond genus level (assignments to “uncultured” were not considered). Most RSVs (ribosomal sequence variants) were assigned to the phyla Proteobacteria, Firmicutes, Cyanobacteria, Actinobacteria, Bacteroidetes, Fusobacteria, Acidobacteria, Deinococcus-Thermus, Planctomycetes and Chloroflexi (in decreasing order). Of these, *Corynebacterium* (8.7%; Actinobacteria), *Ralstonia* (8.5%; Proteobacteria), *Staphylococcus* (6%; Firmicutes), *Acinetobacter* (5%; Proteobacteria), *Streptococcus* (4.8%; Firmicutes), *Pseudomonas* (3.7%; Proteobacteria), *Propionibacterium* (2.6%; Actinobacteria), *Burkholderia* (2%; Proteobacteria), *Moraxella* (1.7%; Proteobacteria), *Prevotella* (1.3%; Bacteroidetes), *Veillonella* (1.2%; Firmicutes) and *Stenotrophomonas* (1.1%; Proteobacteria) showed a relative abundance above 1% of the entire dataset.

### Modules shared a microbial core community, with the highest microbial diversity detected in the utility module EU-250

Alpha diversity analysis of the microbial abundances based on RSVs of each module revealed the highest diversity based on RSVs in the utility module EU-250 (Shannon-Index: 5.4, Additional file 10: Figure S4). The lowest diversity was observed in the medical module EU-100 (Shannon-Index: 4.8). Pairwise comparisons suggested a significant difference (Kruskal-Wallis test) in microbiota composition between the medical module EU-100 and the habitable module EU-150 (*H* = 4.7, *p* = 0.03, *q* = 0.04) and the utility module EU-250 (*H* = 8.3, *p* = 0.004, *q* = 0.01). Analysis of composition of microbiomes (ANCOM; see Additional file 11: Table S6) showed significant differential abundances between the modules for *Actinomyces* (*W* = 480) and *Finegoldia* (*W* = 451). Higher relative percent abundances for *Actinomyces* were detected in the utility module EU-250, while *Finegoldia* was more abundant in the habitable module EU-150. However, contrary to the PhyloChip G3 results, the comparison between the habitable module EU-150 and the utility module EU-250 displayed no significant differences (*H* = 1.3, *p* = 0.2, *q* = 0.2).

This was confirmed by beta diversity level NMDS analysis based on Bray-Curtis distances (stress = 0.07). The NMDS of individual swab samples revealed a cluster of the different modules in the centre of the plot (Additional file 12: Figure S5). This suggests that they share a similar microbial community (Adonis test: *R* ^2^ = 0.07, *P* = 0.001, Additional file 13: Figure S6).

### Different sampling locations showed significant influences on microbial community structure

The analyzed locations covered surfaces situated in wet rooms, a greenhouse, on tables or used for the storage of clothes and office materials. Six of these locations were wooden, and five were stainless steel surfaces in horizontal as well as vertical orientations. In order to identify significant influences originating from surface material and the orientation, we used the pooled samples (which contained mixed locations, materials and surface orientations) as a baseline for drawn comparisons.

Regarding *horizontally and vertically orientated* sampled surfaces, significant differences (Kruskal-Wallis tests) were observed for horizontally vs. mixed (pooled samples; *P* = 0.01, *H* = 6.0), as well as for mixed vs. vertically orientated surfaces (*P* = 0.04, *H* = 4.1) on the level of alpha diversity for Shannon’s diversity (*H*′) (see Additional file 14: Figure S7).

However, no significant differences were detected for other alpha diversity richness metrics like observed OTUs or Faith’s phylogenetic diversity and horizontal versus vertical surfaces at all. On the contrary, beta diversity distances showed significant differences for all surface positions (PERMANOVA: *P* = 0.001, pseudo-*F* = 4.8; see also Additional file 15: Figure S6). ANCOM identified signatures from Sporichthyaceae hgcl clade (*W* = 408) and *Peptostreptococcus* (*W* = 383) as significantly different abundant taxa, which were highly abundant on vertically oriented surfaces.

The microbiota associated to wooden or stainless steel surfaces was significantly different on the level of alpha diversity (Kruskal-Wallis tests: Shannon’s diversity (*H*′) *p* = 0.001, *H* = 10.5; see Additional file 16: Figure S9) as well as beta diversity estimates (PERMANOVA: *p* = 0.001, pseudo-*F* = 7.0; Additional file 17: Figure S10). *Actinomyces* signatures showed significantly higher abundances on stainless steel surfaces compared to low proportions on wooden surfaces (ANCOM: *W* = 486).

In contrast, microbiota from different specific locations inside the modules showed only minor differences (Additional file 18: Figure S11). Larger differences were detected on the level of beta diversity between samples obtained from the toilet bowl or the greenhouse compared to desktop and table surfaces (PERMANOVA: *p* = 0.001, pseudo-*F* = 3.4), which showed that 29% of the variation could be explained by different sampled locations (Adonis: *p* = 0.001, *R* ^2^ = 0.29, Additional file 19: Figure S13). As also identified for surface positions, signatures from Sporichthyaceae hglc clade (ANCOM: *W* = 441) and *Peptostreptococcus* (ANCOM: *W* = 417) as well as *Lachnoanaerobaculum* (ANCOM: *W* = 428) showed significantly different abundance patterns across sample groups. All three taxa were especially high in abundance in samples from wet room associated surfaces (vanity basin and shower cabin faucet).

Fluctuations of the microclimate (i.e. temperature, relative humidity and oxygen and carbon dioxide levels) seemed insufficient to particularly affect the structure of the microbiota since most correlations to microbial compositions for alpha and beta diversity were not significant. Only relative humidity could be significantly correlated with alpha diversity (Spearman rank correlation: *p* = 0.05, Rho = 0.2).

### Community diversity decreased and composition changed over time, potentially influenced by the cleaning regime

We were particularly interested in the change of the microbial community composition over time, i.e. 520 days of confinement. When analyzing the microbial community diversity according to time, a significant, negative correlation was found between Shannon diversity index and day of isolation, suggesting that the microbial community diversity decreased over time (Spearman rank correlation: *p* = − 0.3483, *p* = 0.0003, Additional file 20: Figure S13).

However, the community diversity was fluctuating rather strongly over time. In the beginning of the experiment, between days 14 (06/10) and 44 (07/10) when only medical module EU-100 was sampled (Fig. 2), as well as between 14 (06/10) and 136 (10/10), the median diversity decreased significantly (Kruskal-Wallis pairwise Shannon *p* = 0.049 and *p* = 0.042, respectively), until day 196 (12/10) when the diversity was significantly increased (Kruskal-Wallis pairwise Shannon *p* = 0.017, compared to day 14).

Here, it must be pointed out, that the cleaning regime changed several times and probably influenced the microbial community diversity (and composition; Fig. 2). In the first months, the crew used cleaning solution Katamin AB (a highly effective antimicrobial disinfectant) diluted with pure water to clean all surfaces. However, at around the sampling event on day 196 (12/10), the regime was changed and Katamin AB was only used for metallic surfaces further on.

Around the 253th day of confinement (02/11), the use of Katamin AB was discontinued in all areas and surfaces, and dish washing liquid was used for all cleaning for the rest of the experiment. At days 253 (02/11) and 286 (03/11), the diversity decreased significantly (Kruskal-Wallis pairwise Shannon *p* = 0.039) and is in its lowest point after 8 to 9 months after starting the experiment. This observation might point to active growth of some specific bacteria, suppressing the signatures of inactive or less active species. This effect is then reflected in the abundance distribution and might be an explanation for the decreased diversity. Notably, highest peaks of CFU counts (cultivables) were identified on days 253 (02/11) and 316 (04/11), indicating an impact on the global, molecular and cultivable microbial community, and a potential selective enrichment of certain microbial species.

**Fig. 8:**
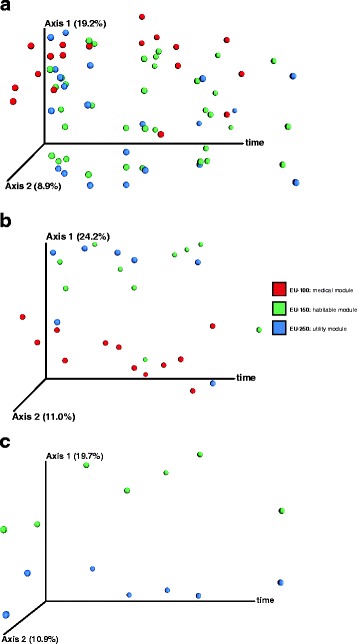
PCoA plot based on Bray-Curtis distances per module over time. X-axis refers to day of isolation. Medical module EU-100 is displayed in red, habitable module EU-150 is shown in green and the utility module EU-250 is highlighted in blue. **a** NGS dataset showing all samples. **b** NGS dataset showing only samples pooled per module and sampling event. **c** PhyloChip dataset of pooled samples per module EU-150 and EU-250 at different sampling events than NGS

Beta diversity analysis showed an increasing distance to the first sampling time-point over time, suggesting that overall the community composition changed significantly during the experiment (PERMANOVA *p* = 0.007; Fig. 8, Additional file 21: Figure S14).

ANCOM confirmed that signatures of six bacterial genera significantly decreased during confinement, based on 50th and 100th percentiles of the RSV distribution. These taxa were *Acidovorax*, *Enterococcus*, *Chroococcidiopsis*, *Pelomonas*, *Staphylococcus* and *Burkholderia*.

### Cultivation retrieved microbial genera that were not detected by molecular methods

**Fig. 9:**
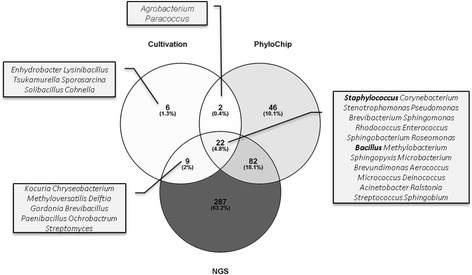
VENN diagram of all detected bacterial genera. For the diagram, all detected genera with a complete taxonomic classification were included (400 for NGS, 152 for PhyloChip and 39 for cultivation). The Venn diagram was prepared using Venny [127]

All three approaches used in this study revealed a different picture of the microbial community present (Fig. 9). It should be noted that Archaea were not detected with any method.

*Staphylococcus* and *Bacillus*, which were found to be the most abundant in cultivation approaches, were detected with all methods independently. However, both genera were not detected as one of the most abundant taxa via PhyloChip nor NGS analysis.

A core microbiota, retrieved from all three methods and consisting of 22 mainly human-associated genera, was identified. Genera that are known to be associated with humans are staphylococci, *Corynebacterium*, *Enterobacter*, *Micrococcus* and *Pseudomonas*. Bacilli, *Aerococcus*, *Methylobacterium* and *Paracoccus* are known as typical environmental microorganisms but have also been described as part of the human microbial community [58–64].

However, each method specifically detected microbial genera that were not found with the other methods, i.e. six genera were solely found by cultivation (Fig. 9; detailed information in Additional file 5: Table S3). NGS data revealed the greatest microbial diversity, as it detected 63.2% of all microbial genera found.

### Functional estimations possibly indicated the increase of opportunistic pathogens, bacteria containing mobile elements and stress-tolerant bacteria over time

**Fig. 10:**
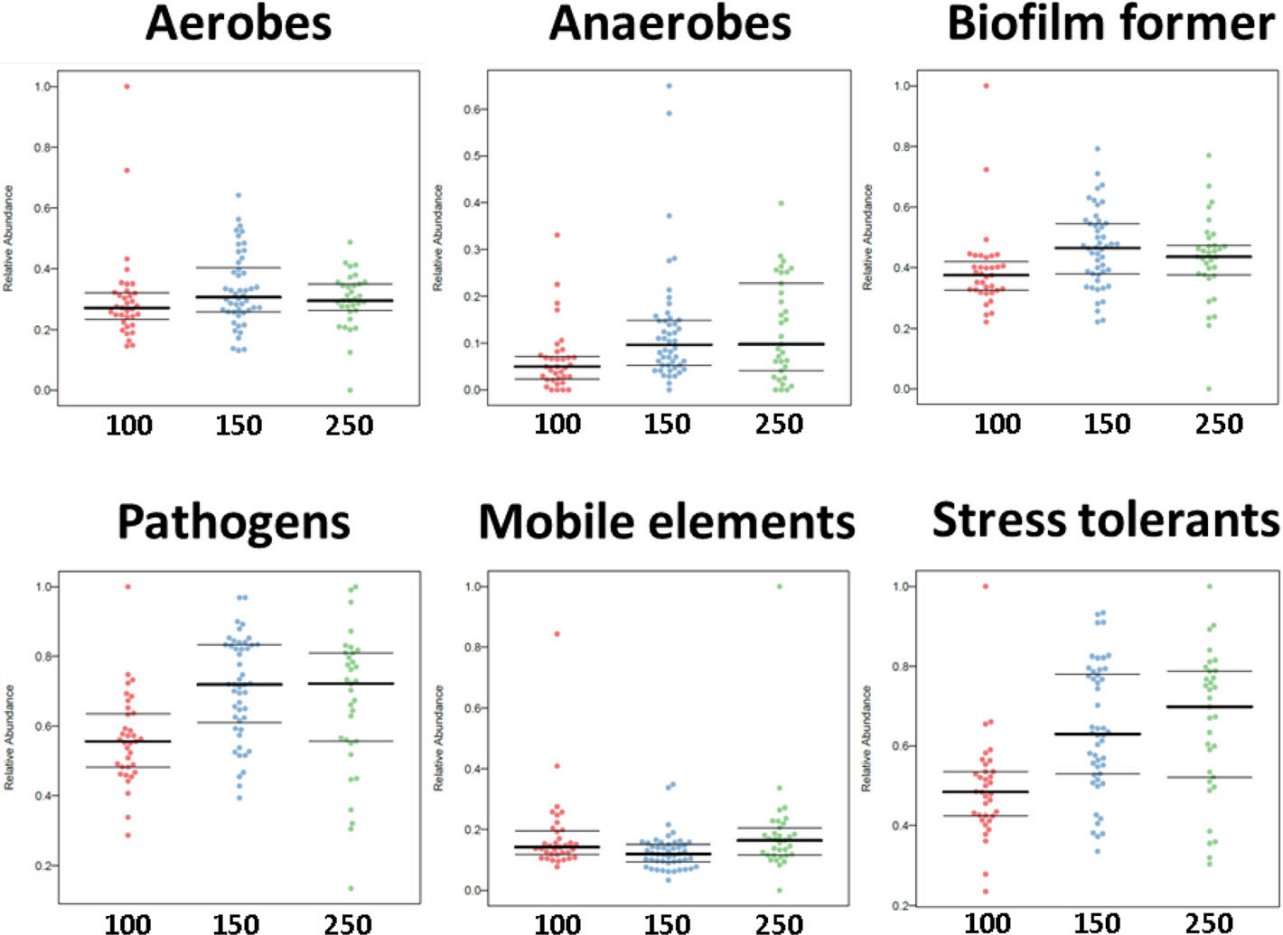
BugBase analyses, based on the NGS dataset. Outcome is grouped according the modules (*x*-axis). The relative abundance is given on the *y*-axis. “Mobile elements” refers to bacteria, most probably carrying mobile elements. Outcome is grouped according the modules EU-100 (“100”), EU-150 (“150”) and EU-250 (“250”; *x*-axis)

As the NGS dataset was found to be the most comprehensive one, it was used for BugBase analyses, allowing a rough prediction of the proportion of e.g. biofilm forming, pathogenic, mobile element containing, oxygen utilizing and oxidative stress tolerant microorganisms ([55]; Fig. 10). As this tool relies only on predicted functional capabilities of assigned taxa from e.g. 16S rRNA gene markers, its ability to capture especially highly dynamic processes like transfer of mobile genetic elements must be considered critically.

The highest abundance of potential pathogens, bacteria with the ability to form biofilms or to tolerate stress, was detected in modules EU-150 (habitable module) and EU-250 (utility module). Differences in relative abundance of pathogens in modules EU-150 and EU-250 towards module EU-100 (medical module) were significant (Kruskal-Wallis test group *p* value = 0.0001, FDR-corrected pairwise Mann-Whitney-Wilcoxon test; *p* values were EU-100 vs. EU-150 *p* = 3.3 × 10^−5^; EU-100 vs EU-250 *p* = 7 × 10^−3^; EU-150 vs. EU-250 *p* = 4.3 × 10^−1^). Within the PhyloChip G3 dataset (due to the full classification to species level), a number of risk group 2 bacteria [65] could be identified, including *Brevibacterium sanguinis*, ***Brevundimonas diminuta***, *Corynebacterium amycolatum*, *Enterobacter hormaechi*, *Enterococcus faecalis*, *Gordonia terrae*, *Klebsiella oxytoca*, *Paracoccus yeei*, ***Roseomonas mucosa***, ***Sphingobacterium multivorum***, ***Staphylococcus aureus***, ***S. epidermidis***, ***S. haemolyticus***, *S. hominis*, *S. lugdunensis*, *S. pettenkoferi*, *Streptococcus salivarius* and *Tsukamurella pulmonis*. The species in bold were also covered by the cultivation approach.

Notably, BugBase predictions on the NGS data potentially indicated the slight increase of signatures from potential pathogenic, stress tolerant microorganisms and those containing mobile elements (Additional file 22: Figure S15). This trend might indicate a potential response of the microbial community towards confinement and was only significant for mobile elements (Spearman rank correlation: *P* = 0.003). However, it has to be noted that despite the fact that different microbial communities mostly have a major pool of the total repertoire of mobile elements in common, the content can become population specific and even can differ in individual species periodically [55].

## Discussion

“Human spaceflight is a complex undertaking that entails numerous technological and biomedical challenges. Engineers and scientists endeavor, to the extent possible, to identify and mitigate the ensuing risks. The potential for an outbreak of an infectious disease in a spacecraft presents one such concern, which is compounded by several components unique to an extraterrestrial environment. Various factors associated with the spaceflight environment have been shown to potentially compromise the immune system of astronauts, increase microbial proliferation and microflora exchange, alter virulence and decrease antibiotic effectiveness. An acceptable resolution of the above concerns must be achieved to ensure safe and efficient space habitation […]. Because many of these clinical concerns are also relevant in terrestrial society, this research will have reciprocal benefits back on Earth” [19; see also 56].

This statement summarizes the urgent need for the understanding of microbial behaviour, dispersal routes, frequencies, associated risks for human health and potential counter strategies in confined environments, such as spacecraft. Aware of this lack of knowledge, we seized the chance to microbiologically analyse the confined, crewed Mars500 habitat, to unveil microbial enumeration and distribution as well as microbiota dynamics over 520 days of isolation.

The most confined habitat available today is the International Space Station [25], which has been constantly inhabited since November 2000. Along with humans, come large numbers of microorganisms, and thus the associated microbial community must to be monitored and if necessary controlled. The allowed thresholds are described in the ISS MORD (Medical Operations Requirements Document, [66]), with 1.0 × 10^4^ CFU/100 cm^2^ being defined as the upper acceptable limit of microbial surface contamination [67, 68]. Notably, reported values which ranged between 25 and 4.3 × 10^4^ CFU/100 cm^2^ of swab samples taken on various surfaces of the ISS have exceeded the anticipated threshold in up to 40% of all analyses [68]. In our study of the Mars500 habitat, the overall mean value of 6.8 × 10^3^ CFU/100 cm^2^ was below the ISS limit, with only 14% of the individual samples exceeding this threshold. Those microbial hotspots were identified mainly in the habitable module, including the exterior of the toilet (13 out of 18 samples) or the table of an individual compartment. Another microbial hotspot was found in the utility module, namely the wall above the vanity basin. Similar values and hotspots were described from a 30-day long confinement experiment at the inflated lunar/Mars analog habitat (ILMAH), where the bedroom was identified as a microbial hotspot [37]. The microbial contamination level in indoor environments is, in general, highly correlated with human presence in the respective area and is also influenced by the type of activity it is used for, such as dining, hygiene, exercise and housekeeping, which leads to a reallocation and/or increase of microbes and nutrients. Typically, each human releases approximately 10^9^ skin cells per day, while coughing or speaking expels between 10^3^ and 10^4^ droplets containing bacteria (sneezing up to 10^6^; [69, 70]). Thus, not only elevated surface microbial contamination is associated with human activity but also the airborne contamination levels reflect the presence of humans, as shown in this study, where highest values were obtained in the individual compartment (2.6 × 10^2^ CFU/m^3^) and in the community room (approximately 1.5 × 10^2^ CFU/m^3^). This finding is in accordance with data from the Mir station revealing that, besides occasional increases due to human physical activity, 95% of analyzed air samples contained less than 5.0 × 10^2^ bacterial CFU/m^3^ (Russian upper limit for piloted space vehicles, [15, 71, 72]). The highest air contamination levels were measured close to the exercise machines on Mir (3.5 × 10^3^ CFU/m^3^). In our study, a comparable low airborne bacterial count of 5.4 × 10^2^ CFU/m^3^ (max. value) was measured next to the treadmill. On board the ISS, allowing maximal 1.0 × 10^3^ bacterial CFU/m^3^ in air, the highest microbial load (7.1 × 10^2^ CFU/m^3^) was encountered in the toilet area (7.1 × 10^2^ CFU/m^3^; [66, 68, 73]).

However, human presence and activity did not only influence the microbial abundance on surfaces and in the air, it also affected the microbiota composition. This is in accordance with previous studies showing that microbial fingerprints of sampled human body parts were resembling those of sampled home surfaces [33]. Cultivation efforts identified mainly human-associated staphylococci, and, in lower abundance, bacilli from all three habitats, which is consistent with findings in periodically confined habitats, such as the ILMAH [37], airplanes [74] and the Antarctica base Concordia [35]. ISS [75] and manned Russian space vehicles [71] also revealed a similar microbial composition, based on cultivation assays. Generally, the high abundance and omnipresence of staphylococci serves as an excellent biomarker for human presence and activity in various indoor environments [37, 76–81]. *Staphylococcus* is a Gram-positive, non-motile bacterium with a wide distribution on skin and in upper respiratory tracts, as well as in soil [82]. Most representatives of this genus are harmless residents on skin and mucous membranes, but as an opportunistic pathogen, staphylococci and particularly the antibiotic resistant strains are known to cause serious infections, especially in hospital environments [83]. Notably, even transmission of *S. aureus* among crew members has been reported [84, 85] and Ilyin [71] claimed an increased incidence of *S. aureus* over time under spaceflight conditions.

Accordingly, the most abundant microbial families in the Mars500 facility were found to represent typical members of the human microbiota as well (PhyloChip analyses, Additional file 7: Figure S2; Lachnospiraceae, Pseudomonadaceae, Ruminococcaceae, Corynebacteriaceae, Comamonadaceae and Rikenellaceae) [86–93]. Therefore, it is not surprising that, for example, eOTUs assigned to Corynebacteriaceae contemporaneously showed an increase during human presence and activity but decreased after the confinement ended. A similar trend has been shown in hospital communities after hospital opening [34].

The majority of found microorganisms and signatures thereof (i.e. 95% of all eOTUs) were assigned to four phyla: Proteobacteria, Firmicutes, Bacteroidetes and Actinobacteria. Predominance of these phyla has been reported from commercial aircraft air filters [94] or cabin air [74].

The abundance of Firmicutes, and to a lesser extent Actinobacteria, resembles findings from other indoor settings mainly retrieved from occupied houses [95], hospitals [96] and dust within houses [97] and offices [98]. A study that aimed to identify household bacterial communities also stated that these four phyla are predominant, although a local geographical pattern was observed regarding the abundance of Firmicutes (more frequent in the toilet) and Proteobacteria (more frequent in the refrigerator; [99]).

Likewise, both Proteobacteria and Firmicutes were frequently detected to high magnitudes on surfaces associated to wet places (toilet, vanity basin and shower) in the Mars500 facility according to NGS data. Moreover, Proteobacteria were also common to the greenhouse and table surfaces. Actinobacteria and Bacteroidetes showed high abundances on both wet places as well as table surfaces. During the whole isolation period, an opposed trend for the abundance of Proteobacteria and Firmicutes together with Actinobacteria was observed. While Proteobacteria were highly abundant at the beginning and end, Firmicutes and Actinobacteria dominated during the isolation period. Regarding the different Mars500 modules, Proteobacteria, Actinobacteria and Cyanobacteria showed highest abundance in the medical module (EU-100). On the contrary, Firmicutes dominated inside the habitable (EU-150) and utility modules (EU-250). However, the only significant differential abundance was observed for Fusobacteria, which increased inside the utility module (EU-250; ANCOM: *W* = 23).

All in all, these results clearly indicate that humans are important dispersal vectors for bacteria that colonize a built environment, with increasing impact in more confined environments, such as the Mars500 facility [96, 100–103].

However, apart from minor changes in the activity levels due to the crew’s weekly schedule, the human influence on the Mars500 facility microbiota can be considered rather constant. Nevertheless, the microbiota was subject to extreme fluctuations, thus indicating the influence of various other parameters.

Typical fluctuation curves, as were seen during the Mars500 experiment, were reported from air samples from hospitals and aircraft cabin air, which also represent highly controlled environments where HEPA filters are installed [74, 104–107]. Since bacteria are not equally distributed in indoor air (i.e. associated to particles) and can appear in clouds depending on the ventilation procedures and the behaviour of the residents [108, 109], the unequal distribution in air might explain the observed fluctuations. Additional other parameters are cleaning regime, humidity, temperature or indigenous dynamics of the microbiome itself, as indicated by the *Bacillus*-*Staphylococcus* antagonistic behaviour revealed by cultivation. Due to various issues, detailed information on the maintenance and climate parameters from the actual sampling day was not available and thus could not be used for detailed assessment of the impact of those parameters on the microbial community. Noteworthy, Chase et al. [110] indicated that the range of climatic variables in indoor settings are restricted to a narrow range, which might be too weak to drive changes of microbial community structure per se. Highly resolved NGS datasets, however, supported the assumption that the cleaning regime had a severe impact on the microbial community found on various surfaces. This finding mirrors earlier reports on the importance of cleaning regimes in cleanrooms or pharmaceutical facilities in order to avoid microbiological outbreaks [111].

The Mars500 microbiome was found to be influenced by a plethora of different factors including the surface material, the location within the facility and/or the function of the respective area. Nevertheless, as shown before [110], it is still questionable if observed differences of the microbial community structure on different surface materials or orientated surfaces are a direct phenomenon or merely a consequence of a distinct interaction behaviour of the marsonauts with different surfaces at certain locations within the Mars500 habitat. Interestingly, sample orientation in the built environment was shown to be a useful indicator of a room’s function [103]. In general, the described dynamics and driving factors of the Mars500 environment support prior findings of the extent and rapidity to which humans passively and actively influence the microbial community of built environments [33, 34, 112]. Van Houdt et al. [35] stated that the higher concentration of Proteobacteria, which was noticed in air samples from the so-called “noisy” part of the Concordia base, might have been a result of the handling with fresh products and vegetables. This might also apply for some eOTUs obtained from the Mars500 samples since the marsonauts grew vegetables in the greenhouse (utility module), whereas food preparation and meals took place in the habitable module. An increase over time was observed in the habitable module for one eOTU that was assigned to *Bifidobacterium*, a probiotic microorganism that is contained in dairy foods and was part of the microbial food supplement during the Mars500 experiment. Therefore, it is not surprising that a greater amount of 16S rRNA gene signatures was mainly found in the habitable module, where samples were taken from the dining table. Along with microbial sequences, organelle signatures from eggplants, peppers, tomatoes, bread wheat and tobacco were significantly differentially abundant (ANCOM: *W* = 6) on table surfaces compared to other locations of the Mars500 habitat.

In essence, the microbiota composition of a certain area mirrored the response to a diverse set of locally present stimuli, resulting in distinct microbiota in the different modules.

Apart from the crew representing the main source for the microbial contamination, we identified the confinement time as the strongest trigger, shaping the microbial diversity and composition. Based on the high-resolution NGS dataset, we were able to recognize a significant decrease of microbial diversity over time, although microbial abundance (number of CFUs) remained more or less at the same level. An opposed trend was observed for the Concordia base, where the contamination level increased during the confinement, but diminished after reopening of the base [35]. The loss in diversity, as observed in the Mars500 facility, could indicate potentially problematic developments within the microbial community, as high diversity is generally associated with system stability and health [113]. Apart from a decrease in diversity, a potential increased proportion of pathogens and stress tolerant microorganisms was predicted for the utility and habitable module. Of note is a study by Ilyin [71], who reported an accumulation of pathogenic bacteria within the first weeks of confinement on board the Mir.

In general, the presence of opportunistic pathogens or signatures thereof was to be expected given that the microbial community was strongly influenced by human-associated microbes. When humans are exposed to stress and extreme environmental conditions, as they would experience during a spaceflight, the immune system is negatively affected, and susceptibility to infection is increased [114, 115]. In parallel, bacteria demonstrate enhanced virulence [116, 117] and less susceptibility to various classes of antimicrobial agents [118, 119] as a result of adaptation processes towards more extreme conditions. To date, serious infections during space travel have been limited to mostly superficial skin infections [21]. Among 742 astronauts, 29 infectious disease incidents in the urinary tract and subcutaneous skin infections were reported [21]. Noteworthy is the occurrence the *Enterococcus* species and signatures within the Mars500 facility. It has been reported that ISS isolates of *Staphylococcus* and *Enterococcus* encoded more resistance genes and possessed higher gene transfer capacities than solates that were obtained from ground-control Concordia station [120]. Strains belonging to *Brevundimonas diminuta,* which was also enriched from the Mars500 facility, have previously been enriched from the Mir space station and from clinical settings, where they have been implicated in opportunistic infections [72, 121, 122].

Although certain potentially opportunistic pathogens were cultivated, and the resistance and pathogenic potential was predicted to increase over time, our data are limited by using cultivation efforts and 16S rRNA sequencing data only. Further investigation, for example, on the pathogenic potential of the myriad of isolates obtain would allow an improved risk assessment and an immediate impact of those bacteria on the crew health. However, based on the profound knowledge obtained and accordance of the cultivation data with limits stated in the ISS MORD document, we presume that the marsonauts were not exposed to an increased health risk. This is underlined by the study of Roda et al. [123], which reports the continuous monitoring of the health status of the crew member during the Mars500 isolation experiment. By the use of non-invasive panel tests for gastrointestinal motility investigation, such as via periodic blood biochemical function tests and clinical examinations, the researchers reported that no significant pathology or physiological alteration appeared. In addition, metagenomics analyses of the intestinal microbiome of the marsonauts revealed functional stability over time, although the microbial gut community reflected the environmental changes and underwent a community-wide modification, without any negative impact on the health of the participants [124, 125].

## Conclusion

The applied sampling and processing scheme facilitated the identification of hotspots of microbial accumulation. Overall, an average microbial load that did not exceed the allowed limits for ISS in-flight requirements was observed, which reflects the adequate maintenance of the facility. The findings herein clearly indicate that, under confined conditions, the community structure is still a very dynamic system which adapts to the prevailing habitat and micro-conditions. These results implicate the necessity to screen comprehensively, since results varied from place to place, from surface to surface, and from time to time in terms of quantity and composition of bacterial contaminants.

These dynamics need to be monitored, and under certain circumstances, countermeasures are required to avoid development of highly resistant or potentially pathogenic microorganisms, as well as the accumulation of a few flourishing taxa which might lead to a measurable decrease of microbial diversity. Since a sterile environment is not achievable, it is important to maintain the microbial balance of beneficial, neutral and harmful bacteria for the sake of the system’s stability and health.

## Additional files

Additional file 1: Doc S1. Detailed description of the Mars500 habitat. (DOCX 150 kb)

Additional file 2: Figure S1. Detailed schematic drawing of the three sampled modules of the Mars500 facility A: Utility module EU-250, B: Habitable module EU-150, C: Medical module EU-100; 1A-C: Air sampling locations: Photographs of each sampling site are allocated to the specific location within the habitat, 2A-C Surface sampling locations: Photographs of each sampling site are allocated to the specific location within the habitat. ©photo IMBP/Oleg Voloshin (approved and edited). (PDF 639 kb)

Additional file 3: Table S1. Total CFUs retrieved from 10 cm^2^ surface and the corresponding percentage of the CFUs formed by heat-shock resistant (80 °C, 15 min) bacteria appearing on R2A plates after 72 h incubation at 32 °C. Different locations of the utility module EU-250, the habitable module EU-150 and the medical module EU-100 were sampled. (XLSX 15 kb)

Additional file 4: Table S2. Total microbial airborne CFU counts per 500 l sampled air on R2A after 72 h incubation at 32 °C. Different locations in the utility module EU-250, the habitable module EU-150, and the medical module EU-100 were sampled. (XLSX 13 kb)

Additional file 5: Table S3. List of isolates obtained from surface and air samples obtained from different sampling locations within the three sampled modules: utility module EU-250, the habitable module EU-150, and the medical module EU-100. (XLSX 69 kb)

Additional file 6: Table S4. HybScores of the analysed sampled using PhyloChip. Sheet 1 gives presence and absence values per eOTU and sample. Sheet 2 gives abundance values per eOTU and sample. Indicated are sampling location and date. (XLSX 312 kb)

Additional file 7: Figure S2. Proportional abundance based on aggregated HybScores on family level across selected points in time. Nine families with the largest sum of HybScores from the OTUs within each family are displayed. Staphylococcaceae represent in all analyzed PhyloChip arrays less than 1% of the overall diversity. Asterisk denotes unclassified proteobacterial families. (PDF 23 kb)

Additional file 8: Figure S3. Heatmap of eOTUS that showed a significant correlation (Spearman rank correlation, *p*-value <0.05) with factor time in one module A: Habitable module EU-150, B: Utility module EU-250. The eOTUs are ordered by positive (pink) and negative (cyan) correlation and by p-value in increasing manner. Numbers indicate HybScores. (PDF 646 kb)

Additional file 9: Table S5. Sequences and features of the NGS dataset and summary of statistical tests performed on different alpha diversity metrics, beta diversity and the weighted unifrac metric of the NGS dataset. (XLSX 24 kb)

Additional file 10: Figure S4. Box and whisker plots of the Shannon diversity index of the NGS dataset according to different modules of the Mars500 habitat. (PDF 144 kb)

Additional file 11: Table S6. Summarizing results of differential abundant taxa of the NGS dataset using the ANCOM test. (XLSX 10 kb)

Additional file 12: Figure S5. NMDS of a total 1810 features obtained from the medical module EU-100, the habitable module EU-150 and the utility module EU-250 A) only individual swab samples *n* = 81; B only pooled samples *n* = 37 were considered; stress = 0.007. (PDF 200 kb)

Additional file 13: Figure S6. Showing box and whisker plots of weighted unifrac distances of the NGS dataset according to different modules of the Mars500 habitat. (PDF 176 kb)

Additional file 14: Figure S7. Showing box and whisker plots of the Shannon diversity index of the NGS dataset according to different positions (surface orientations) of the Mars500 habitat. (PDF 106 kb)

Additional file 15: Figure S8. Showing box and whisker plots of weighted unifrac distances of the NGS dataset according to different positions (surface orientations) of the Mars500 habitat. (PDF 189 kb)

Additional file 16: Figure S9. Showing box and whisker plots of the Shannon diversity index of the NGS dataset according to different sampled materials of the Mars500 habitat. (PDF 105 kb)

Additional file 17: Figure S10. Showing box and whisker plots of weighted unifrac distances of the NGS dataset according to different sampled materials of the Mars500 habitat. (PDF 164 kb)

Additional file 18: Figure S11. Showing box and whisker plots of the Shannon diversity index of the NGS dataset according to different locations of the Mars500 habitat. (PDF 139 kb)

Additional file 19: Figure S12. Showing box and whisker plots of weighted unifrac distances of the NGS dataset according to different locations of the Mars500 habitat. (PDF 213 kb)

Additional file 20: Figure S13. Showing box and whisker plots of the Shannon diversity index of the NGS dataset according to the day of isolation (time). (PDF 134 kb)

Additional file 21: Figure S14. Showing box and whisker plots of weighted unifrac distances of the NGS dataset according to the day of isolation (time). (PDF 169 kb)

Additional file 22: Figure S15. BugBase analyses, based on the NGS dataset. Outcome is grouped according the time-point of sampling (“Day of isolation”, x-axis). The relative abundance is given on the y-axis. “Mobile Elements” refers to bacteria, most probably carrying mobile elements. (PDF 95 kb)

## Abbreviations

16S rRNA: Small subunit of ribosomal ribose nucleic acid
ANCOM: Analysis of composition of microbiomes
ANOSIM: Analysis of Similarity
ARBEX: Archaeal and Bacterial Extremophiles on board the ISS
CFU: Colony-forming unit
DNA: Deoxyribose nucleic acid
eOTU: Empirical operational taxonomic unit
ESA: European Space Agency
EU: Experimental unit
FDR: False discovery rate
Hi-SEAS: Hawai’i Space Exploration Analog and Simulation
HS: Heat shock
IBMP RAS: Institute for Biomedical Problems Russian Academy of Sciences
ILMAH: Inflatable lunar/Mars analogous habitat
ISS: International Space Station
iTOL: Interactive Tree Of Life
JAXA: Japan Aerospace Exploration Agency
KEGG: Kyoto Encyclopedia of Genes and Genomes
MICHA: MIcrobial ecology of Confined Habitats and humAn health
MORD: Medical Operations Requirements Document
MRPP: Multi-response permutation procedure
NASA: National Aeronautics and Space Administration
NGS: Next generation sequencing
NMDS: Non-metric multidimensional scaling
PATRIC: Pathosystems Resource Integration Center
PBS: Phosphate-buffered saline
PCoA: Principal Coordinate Analysis
PCR: Polymerase chain reaction
PICRUSt: Phylogenetic Investigation of Communities by Reconstruction of Unobserved States
RSV: Ribosomal sequence variants
