# Supplementary material for: Preparing for the crewed Mars journey: microbiota dynamics in the confined Mars500 habitat during simulated Mars flight and landing
Source: Microbiome. 2017 Oct 4;5:129. doi: 10.1186/s40168-017-0345-8 (PMC5627443; doi:10.1186/s40168-017-0345-8)
Supplement: Supplementary file 1 — Doc S1. Detailed description of the Mars500 habitat. (DOCX 150 kb) [file 40168_2017_345_MOESM1_ESM.docx]

**Technical data of modules**

In all habitable modules a life support system was installed, consisting of a gas analysis system, air-conditioning and ventilation system, sewage system, water supply, as well as a fire alarm and suppression system.

**Module EU-100 – Medical module**

Volume: 100 m³

Size of the floor area: 3.2 x 11.9 m²

Purpose: conduction of medical and psychological experiments

Inventory: This module housed two medical berths, a toilet, and equipment for routine medical examinations and telemedical, laboratory and diagnostic investigations. In case a marsonaut would have been ill, he would have been isolated and treated there. However, this module was frequented more often than planned since the bed located there was bigger than the ones in the individual compartments (pers. comment: marsonaut).

Passage from EU-100 to EU-250, and EU-50 was not accessible directly, since only one transfer tunnel was built that connected EU-100 with EU-150. At one end of the module a hermetical door was installed whereas an emergency door was located at the opposite site.

**Module EU-150 – Habitable module**

Volume: 150 m³

Size of the floor area: 3.6 x 20.0 m²

Purpose: main living quarters for the crew

Inventory: The module housed six individual compartments for the crewmembers, a kitchen-dining room, a living room, the main control room, and a toilet. The individual compartments (bedrooms 2.8 - 3.2 m^2^) had a bed, a desk, a chair, and shelves for personal belongings.

EU-150 possessed three hatches and transfer tunnels that were hermetically sealed, but providing the possibility to reach every experimental unit directly.

**Module EU-250 – Utility module**

Volume: 250 m³

Size of the floor area: 3.9 x 24.0 m²

Purpose: storage of food, clothes, supplies, as well as location of the fitness area and the greenhouses

Inventory: This module was divided into compartments consisting of a fridge for storage of food, a compartment for storage of non-perishable food, experimental greenhouse, the bathroom, sauna, and gym.

The technical installations included all necessary equipment for running the study (communications and control, ventilation and air supply, water supply, electrical installations, sewerage, air and water quality monitoring and partial recycling, medical equipment, fire and other safety monitoring systems, emergency equipment, etc.). Waste was disposed through lock chambers.

One hermetical door was installed to connect to module EU-150 via a transfer tunnel. Two additional hermetical doors with stairs were located at both ends of the module for loading the complex with supply material in advance.

**Module EU-50 – Simulator of the landing Martian ship**

Volume: 50 m³

Size of the floor area: 6.3 x 6.17 m²

Purpose: simulation of the Martian landing module for three crew members, only used during the 30 days long 'Mars orbiting' phase

Two transfer tunnels with hatches allowed passing into module EU-150 and into the lock of the chamber of the simulator of the Martian surface.

**External Module SMS (Simulator of the Martian surface)**

Purpose: simulation of the Martian surface

This module included a non-hermetical chamber. The transfer tunnel with stairs (hermetically sealed) connected the SMS module with EU-50.

**Life support system**

The life support system consisted of four independent systems (one in each module) that guaranteed the autonomy of each module; all four included main and auxiliary systems to ensure a long-term stay for people in this closed environment. The crew stayed in those modules under an artificial atmospheric environment at normal barometric pressure. The main system controlled module climate, gas supply and maintenance of the indoor atmosphere, purification of the atmosphere, water supply and sewerage, video monitoring, supply of electric power, communication, and information. The auxiliary system included following equipment: a computer-aided system for collection and reflection of parameters within the inhabited zone, a thermoregulation system, a dehumidification system for removing condensate from the atmospheric moisture, and a system for climate control.

**Air control system and water processing unit**

The following section provides more details regarding the indoor air control system as well as about the drinking water and liquid waste water processing unit to understand and interpret the obtained results. Maintenance of the required air quality within the respective four experimental facilities (EU-50, EU-100, EU-150, and EU-250) was realized by independently operating air conditioning systems. Either the external “ground-based” control crew or the marsonauts themselves were allowed to regulate the habitat’s environmental conditions within specified limits. Readings about the current environmental data were taken from screens installed in each module.

Each system was equipped with a ceiling-mounted tunnel air conditioning system made by Wolf GmbH (Mainburg, Germany), which collected the moisture by use of a hermetic cover. The air channels were equipped with gates to prevent airflow; the latter were mounted at the inlet and outlet points of the air conditioner, as well as in the air ducts inside the experimental units in the sub-ceiling of the rooms. The purification of the atmosphere was performed at the pilot plant, and the stagnant air was replaced by use of two carbon filters and an additional filter containing calcium carbonate to absorb carbon dioxide. The replacement of the filter cartridges should be performed on average every 36 h. The allowed relative concentration of carbon dioxide in the atmosphere should be within the limits of 0.1 to 0.6 %. The air flow rate through the filter systems was carried out by a fan and was regulated by the duty engineer; it should be within the limits of 50 to 300 m³ per h.

To remove dust, microorganisms and fungi from the atmosphere, an industrial photocatalytic system, AIRcomfort AC-3020 (Namyangju-City, Kyonggi, Korea), with replaceable filters (figure below) and the Potok 150-M-01 (Potok Inter, Moscow, Russia) were installed. These units were placed inside the module and were serviced by the crew. The flow rate could be adjusted to high (320 m^3^/h) or turbo (440 m^3^/h).


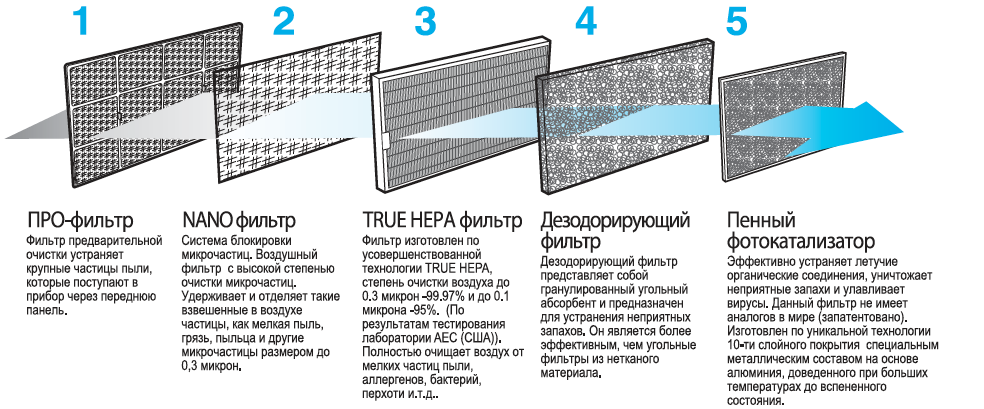


**Fig.** Schematic drawing of the series connection containing five filters with different properties to improve air quality and remove airborne matter. 1: Pro filter; 2:NANO filter; 3:improved TRUE HEPA filter; 4:Deo filter; 5:Foamy photolytic catalyst © 2004-2014 КиМ Онлайн

Components of the industrial photocatalytic system are outlined as follows:

Pro filter

The pre-cleaning filter holds back the large particles of dust which pass through the front panel to the filter.

Cleaning of the front panel is strongly recommended every month or in shorter periods depending on the amount of dust present in a room.

2. NANO filter

Blocking system for the passage of microparticles. Air filter with a high purification level for microparticles >0.3 µm keeping back fine dust, dirt, and pollen.

Changing of the filters should be performed every three months according to the manufacturer’s instructions.

3. Improved TRUE HEPA filter

The filter cleaned the air near-completely from fine dust particles, allergens, bacteria, danders, *et cetera*. Removal of particles <0.3 µm (99.97 % efficiency rate) and matter of size <0.1 µm including microorganisms (95 % efficiency rate according to results of the AEC Laboratories, USA) was achieved.

Exchange of the TRUE HEPA filter, the UV lamp, and the deo filter should be performed in order annually; this task is indicated on the screen of the unit.

4. Deo filter

Disinfection filter consisted of a granular coal absorbent (higher efficiency than fabric filters) that removed bad odors from the air.

5. Foamy photolytic catalyst

Catalyst performed effective removal of organic compounds, odours, and intercepts viruses. This filter is unique based on a 10-fold coating with a special aluminium metal mixture.

Furthermore, each of the modules (EU-50, EU-100, EU-150, and EU-250) was equipped with a system for disposal of household water, potable water supply, and the sewerage system for the removal of food waste and for disposal of excreta of the crew.

The potable water supply system made of stainless steel pipes was designed to provide purified drinking water used for beverages and food. The cold water of the urban water supply flowed through the input unit, which was equipped with the input filter for coarse purification and went on into water tanks that were intended for toilets and showers as well as for the system of post-purification. The tap water was integrated in the cleaning cycle and sanitized by addition of ionized silver, installation of an additional filter set and a UV lamp, to reach the mandatory purification level. Exchange vessels were used for removal of dissolved heavy metals.

The sanitation of the lavatory flush, the sinks of the experimental facilities and the showers was conducted through the sewerage system. Removal of sewage was accomplished by passing the following stations: a storage tank equipped with a membrane - a toilet bowl (basin) - gate valve with electric drive - storage container - fecal pump - valve with electric drive - dumping into the municipal sewage system. The configuration of the above-mentioned structured scheme eliminated the direct connection to the outdoor atmosphere.
