# Supplementary figures and images for: Preparing for the crewed Mars journey: microbiota dynamics in the confined Mars500 habitat during simulated Mars flight and landing
Source: Microbiome. 2017 Oct 4;5:129. doi: 10.1186/s40168-017-0345-8 (PMC5627443; doi:10.1186/s40168-017-0345-8)

## Air sampling

## Surface sampling

1A

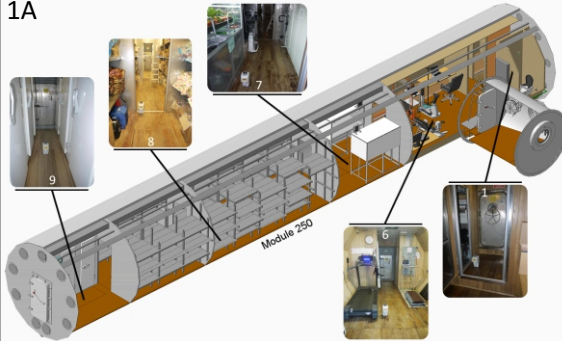

2A

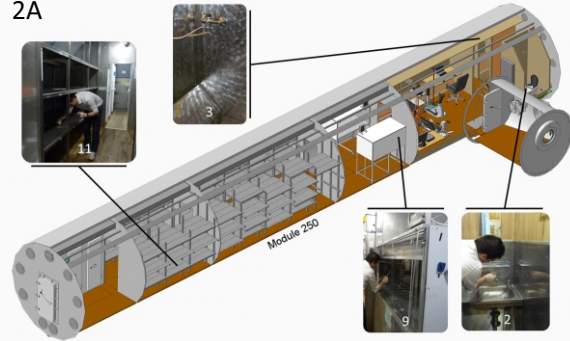

1B

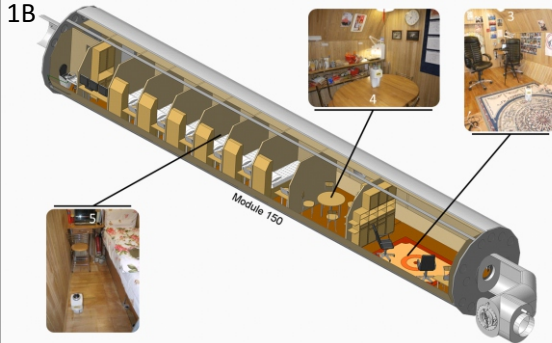

2B

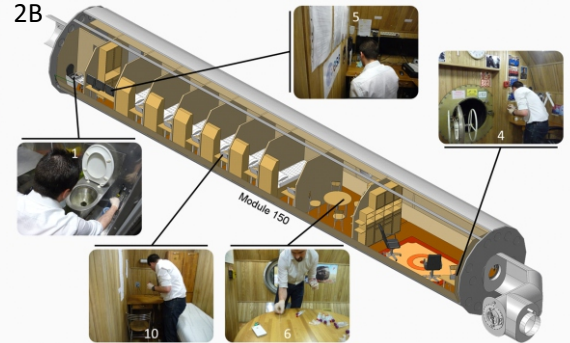

1C

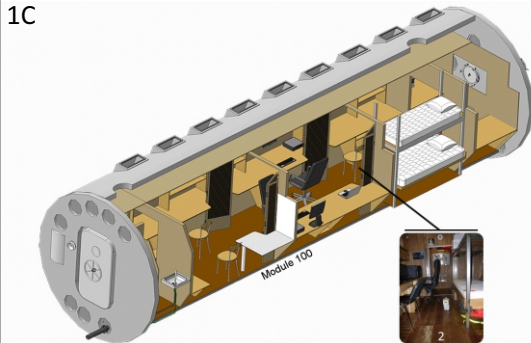

2C

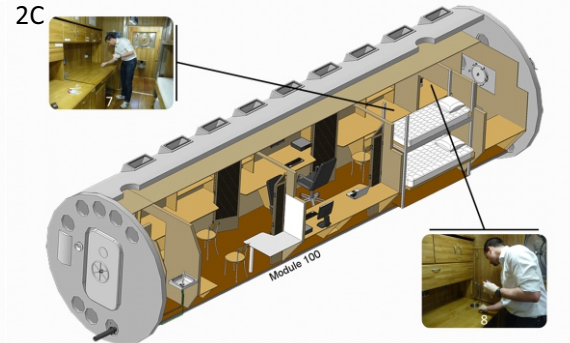

Supplement: Supplementary file 2 — Detailed schematic drawing of the three sampled modules of the Mars500 facility A: Utility module EU-250, B: Habitable module EU-150, C: Medical module EU-100; 1A-C: Air sampling locations: Photographs of each sampling site are allocated to the specific location within the habitat, 2A-C Surface sampling locations: Photographs of each sampling site are allocated to the specific location within the habitat. ©photo IMBP/Oleg Voloshin (approved and edited). (PDF 639 kb) [file 40168_2017_345_MOESM2_ESM.pdf]

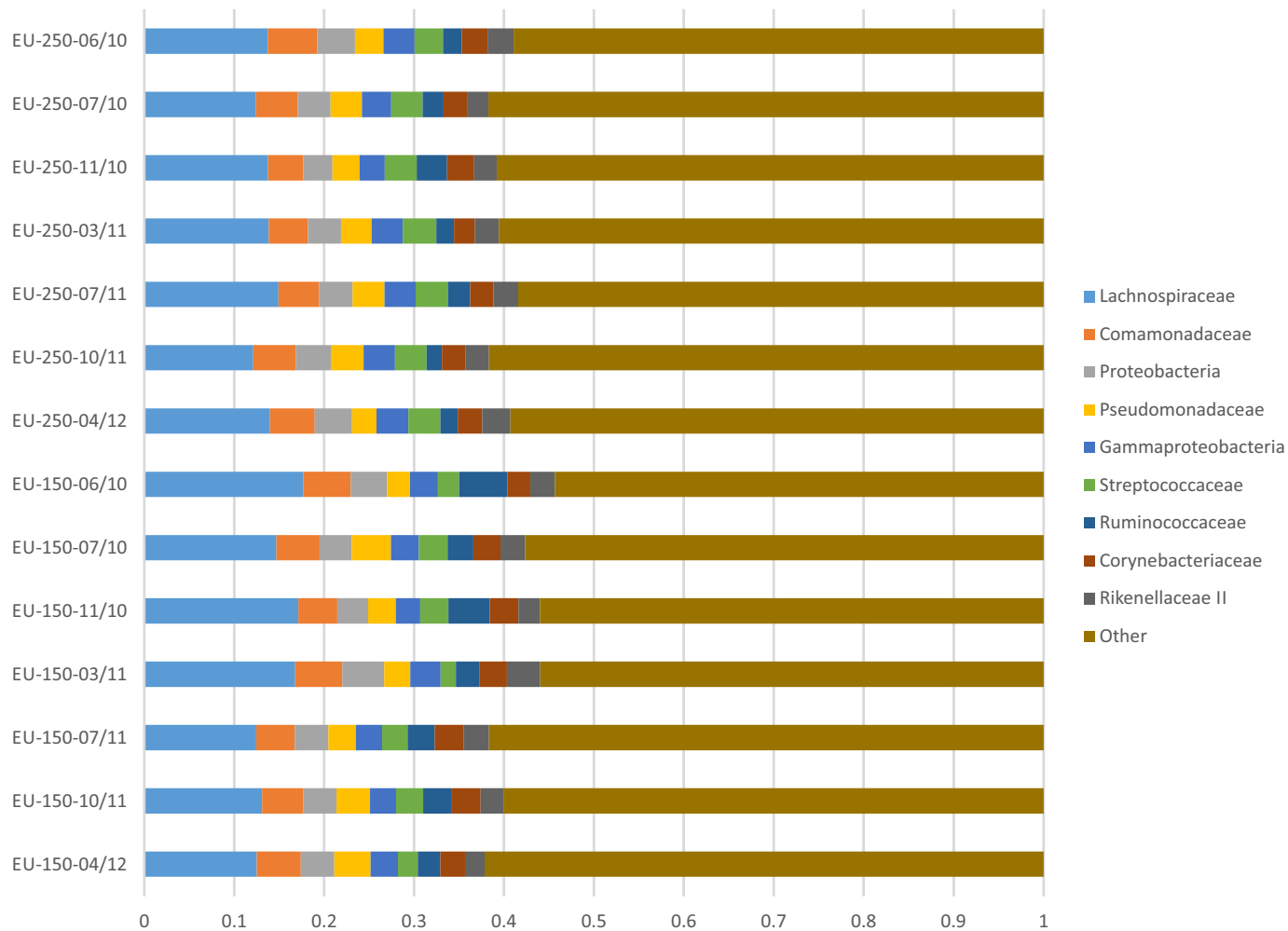

Supplement: Supplementary file 7 — Proportional abundance based on aggregated HybScores on family level across selected points in time. Nine families with the largest sum of HybScores from the OTUs within each family are displayed. Staphylococcaceae represent in all analyzed PhyloChip arrays less than 1% of the overall diversity. Asterisk denotes unclassified proteobacterial families. (PDF 23 kb) [file 40168_2017_345_MOESM7_ESM.pdf]

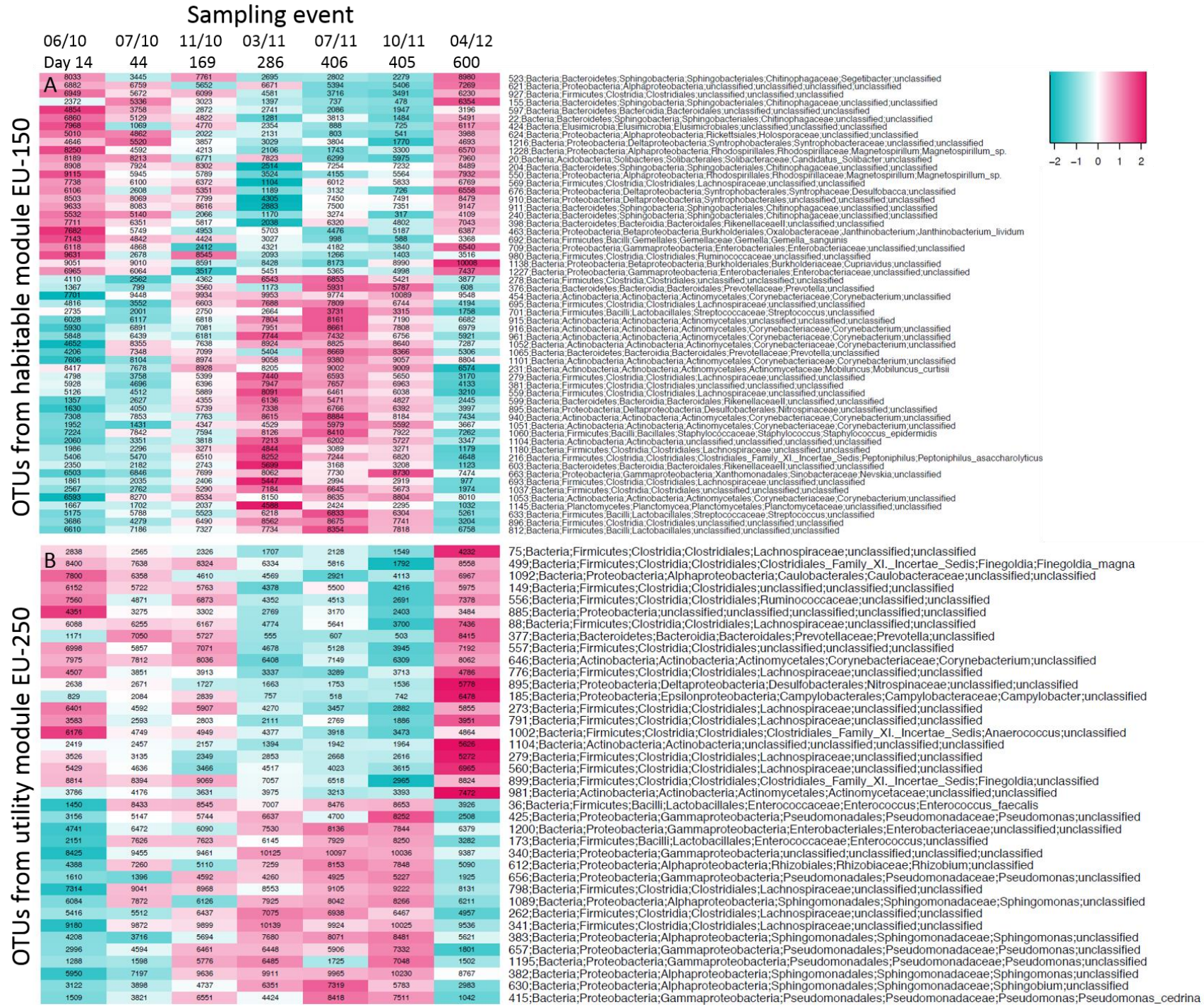

Supplement: Supplementary file 8 — Heatmap of eOTUS that showed a significant correlation (Spearman rank correlation, p-value <0.05) with factor time in one module A: Habitable module EU-150, B: Utility module EU-250. The eOTUs are ordered by positive (pink) and negative (cyan) correlation and by p-value in increasing manner. Numbers indicate HybScores. (PDF 646 kb) [file 40168_2017_345_MOESM8_ESM.pdf]

shannon diversity (H')

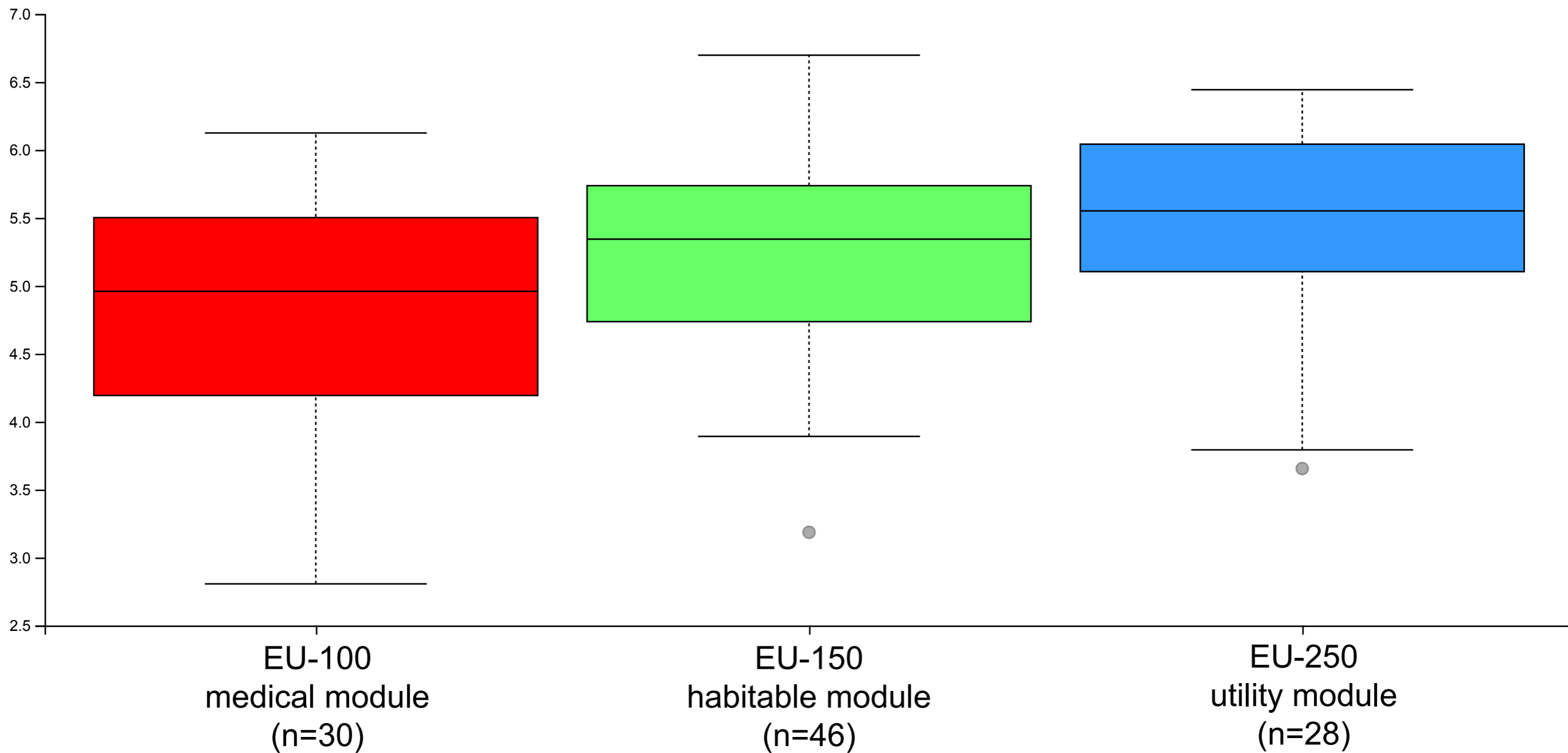

**Mars500 habitat modules**

Supplement: Supplementary file 10 — Box and whisker plots of the Shannon diversity index of the NGS dataset according to different modules of the Mars500 habitat. (PDF 144 kb) [file 40168_2017_345_MOESM10_ESM.pdf]

A

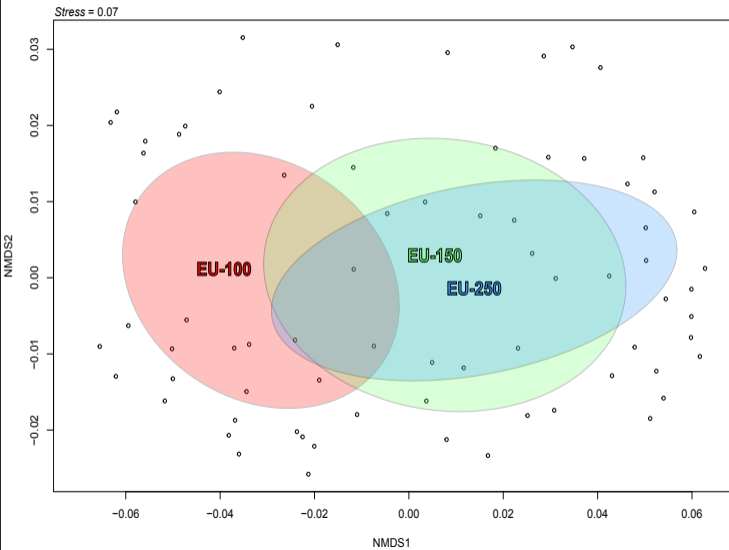

B

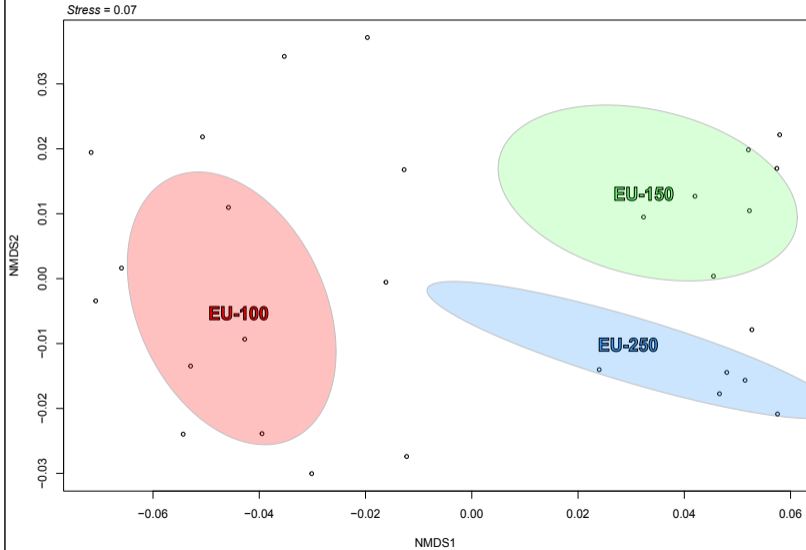

Supplement: Supplementary file 12 — NMDS of a total 1810 features obtained from the medical module EU-100, the habitable module EU-150 and the utility module EU-250 A) only individual swab samples n = 81; B only pooled samples n = 37 were considered; stress = 0.007. (PDF 200 kb) [file 40168_2017_345_MOESM12_ESM.pdf]

**beta diversity distances to the medical module (EU-100)**

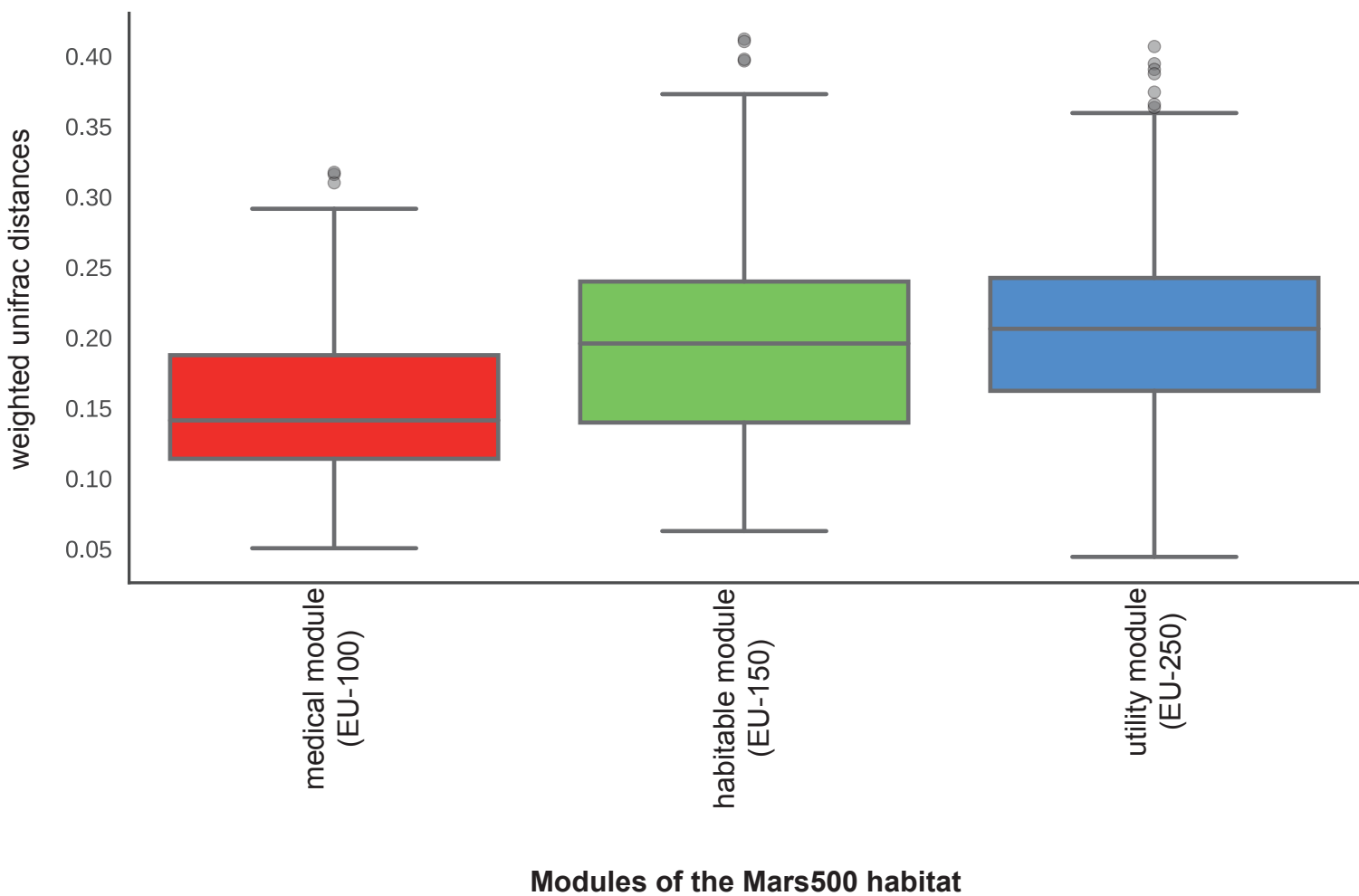

Supplement: Supplementary file 13 — Showing box and whisker plots of weighted unifrac distances of the NGS dataset according to different modules of the Mars500 habitat. (PDF 176 kb) [file 40168_2017_345_MOESM13_ESM.pdf]

Shannon diversity (H')

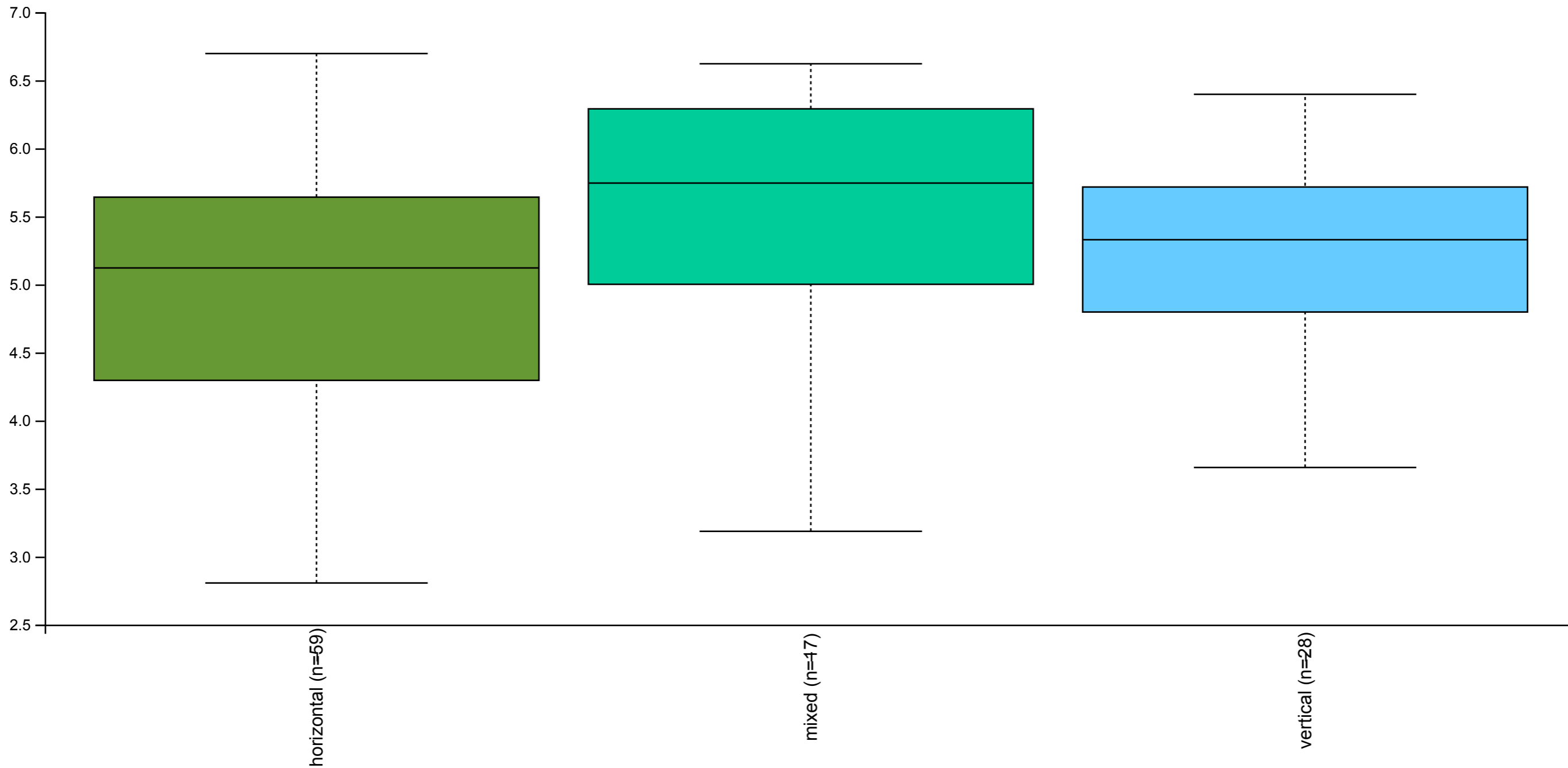

Surface Position

Supplement: Supplementary file 14 — Showing box and whisker plots of the Shannon diversity index of the NGS dataset according to different positions (surface orientations) of the Mars500 habitat. (PDF 106 kb) [file 40168_2017_345_MOESM14_ESM.pdf]

Shannon diversity ( $H'$ )

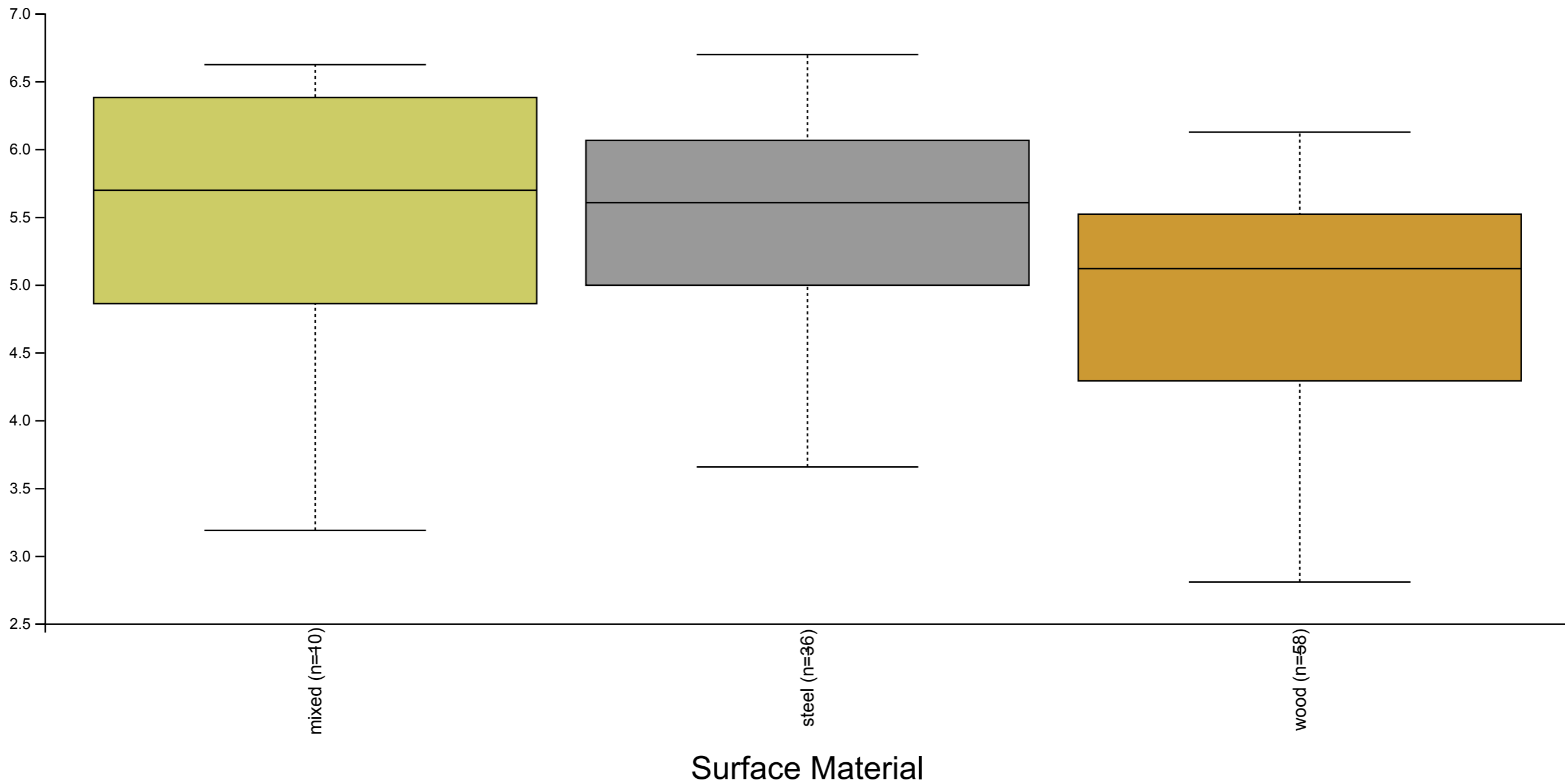

Supplement: Supplementary file 16 — Showing box and whisker plots of the Shannon diversity index of the NGS dataset according to different sampled materials of the Mars500 habitat. (PDF 105 kb) [file 40168_2017_345_MOESM16_ESM.pdf]

# beta diversity distances to samples with mixed material properties

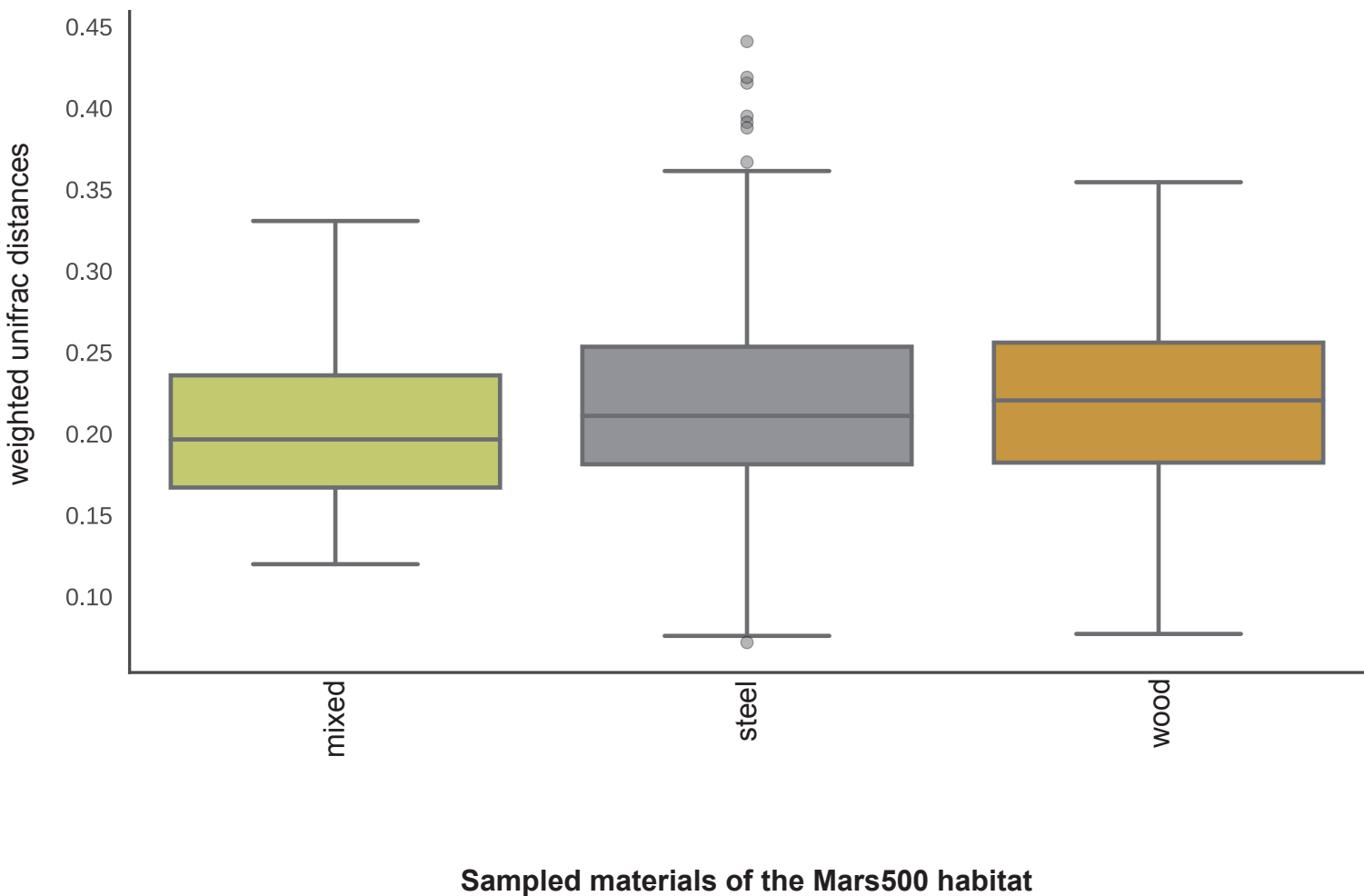

Supplement: Supplementary file 17 — Showing box and whisker plots of weighted unifrac distances of the NGS dataset according to different sampled materials of the Mars500 habitat. (PDF 164 kb) [file 40168_2017_345_MOESM17_ESM.pdf]

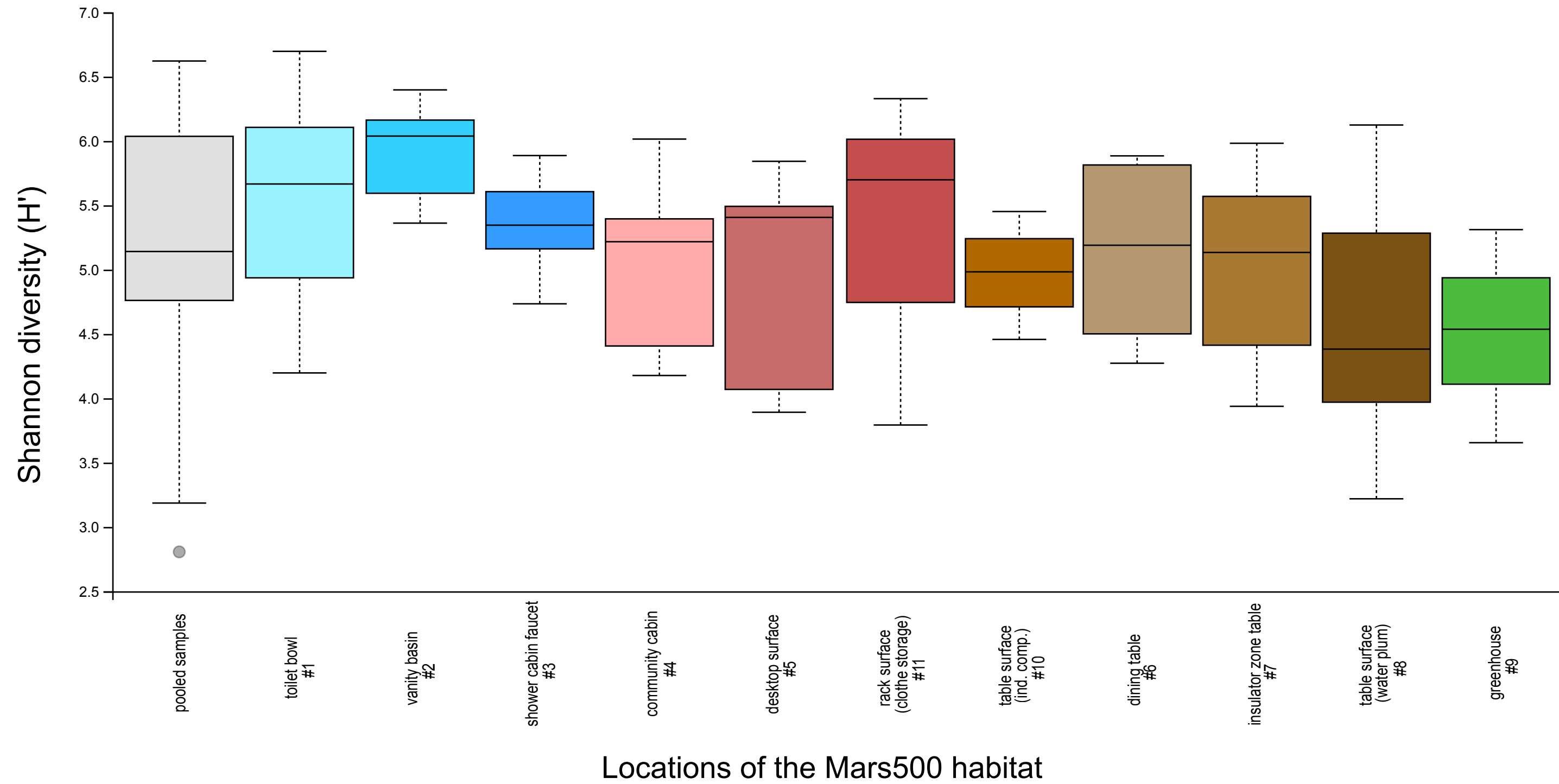

Supplement: Supplementary file 18 — Showing box and whisker plots of the Shannon diversity index of the NGS dataset according to different locations of the Mars500 habitat. (PDF 139 kb) [file 40168_2017_345_MOESM18_ESM.pdf]

# beta diversity distances to pooled samples

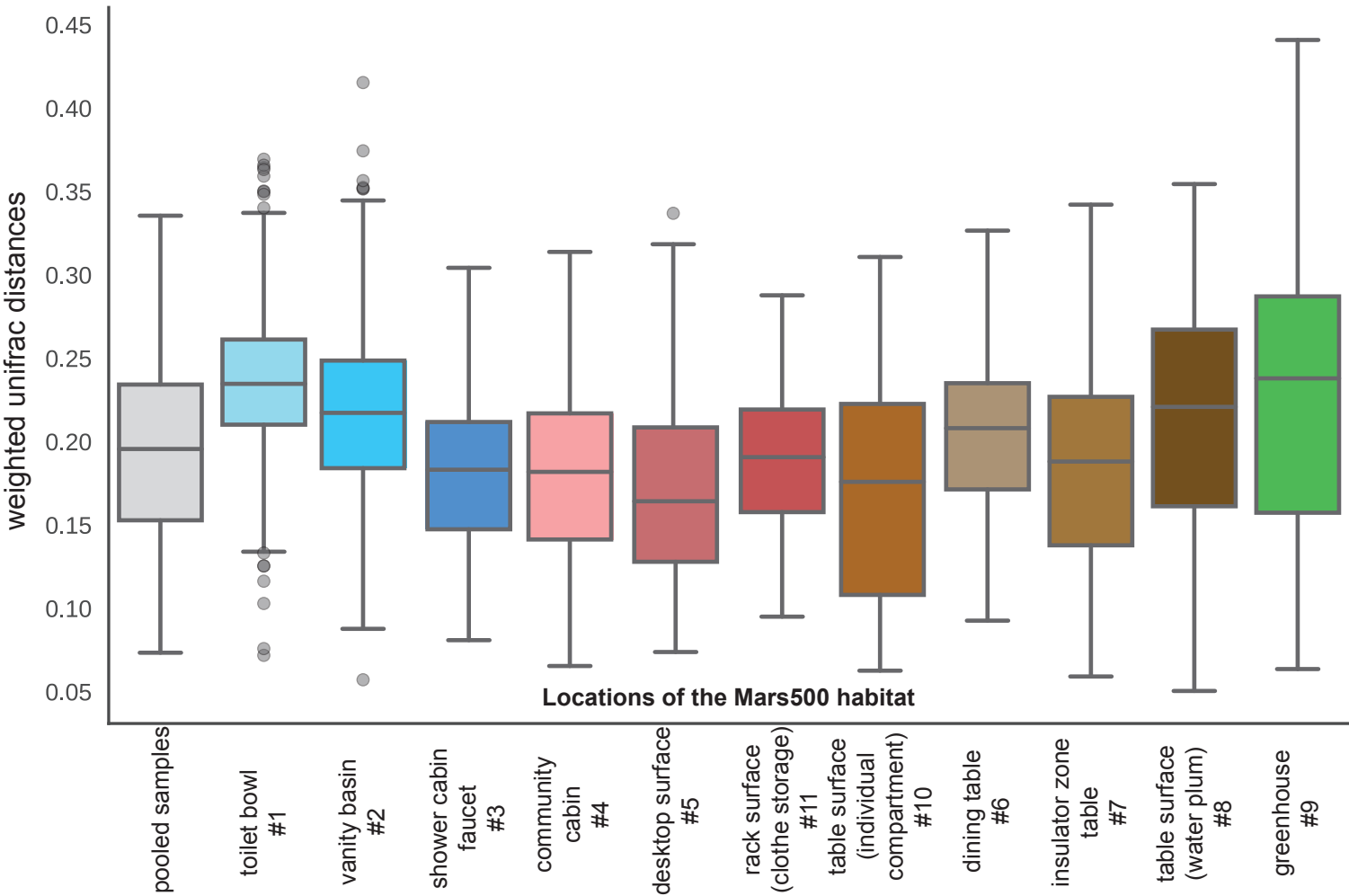

Supplement: Supplementary file 19 — Showing box and whisker plots of weighted unifrac distances of the NGS dataset according to different locations of the Mars500 habitat. (PDF 213 kb) [file 40168_2017_345_MOESM19_ESM.pdf]

Shannon diversity (H')

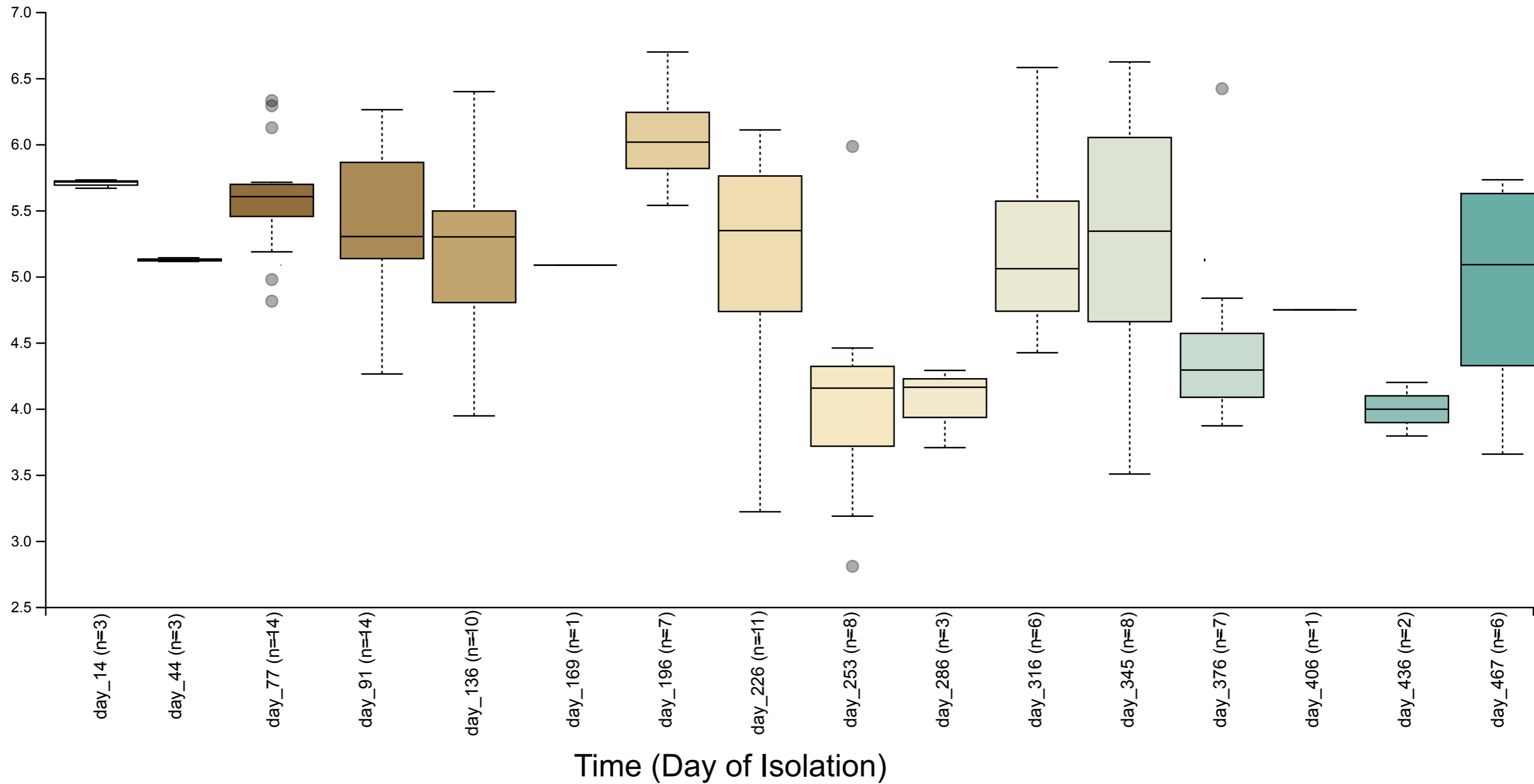

Supplement: Supplementary file 20 — Showing box and whisker plots of the Shannon diversity index of the NGS dataset according to the day of isolation (time). (PDF 134 kb) [file 40168_2017_345_MOESM20_ESM.pdf]

**beta diversity distances to day 14**

weighted unifrac distances

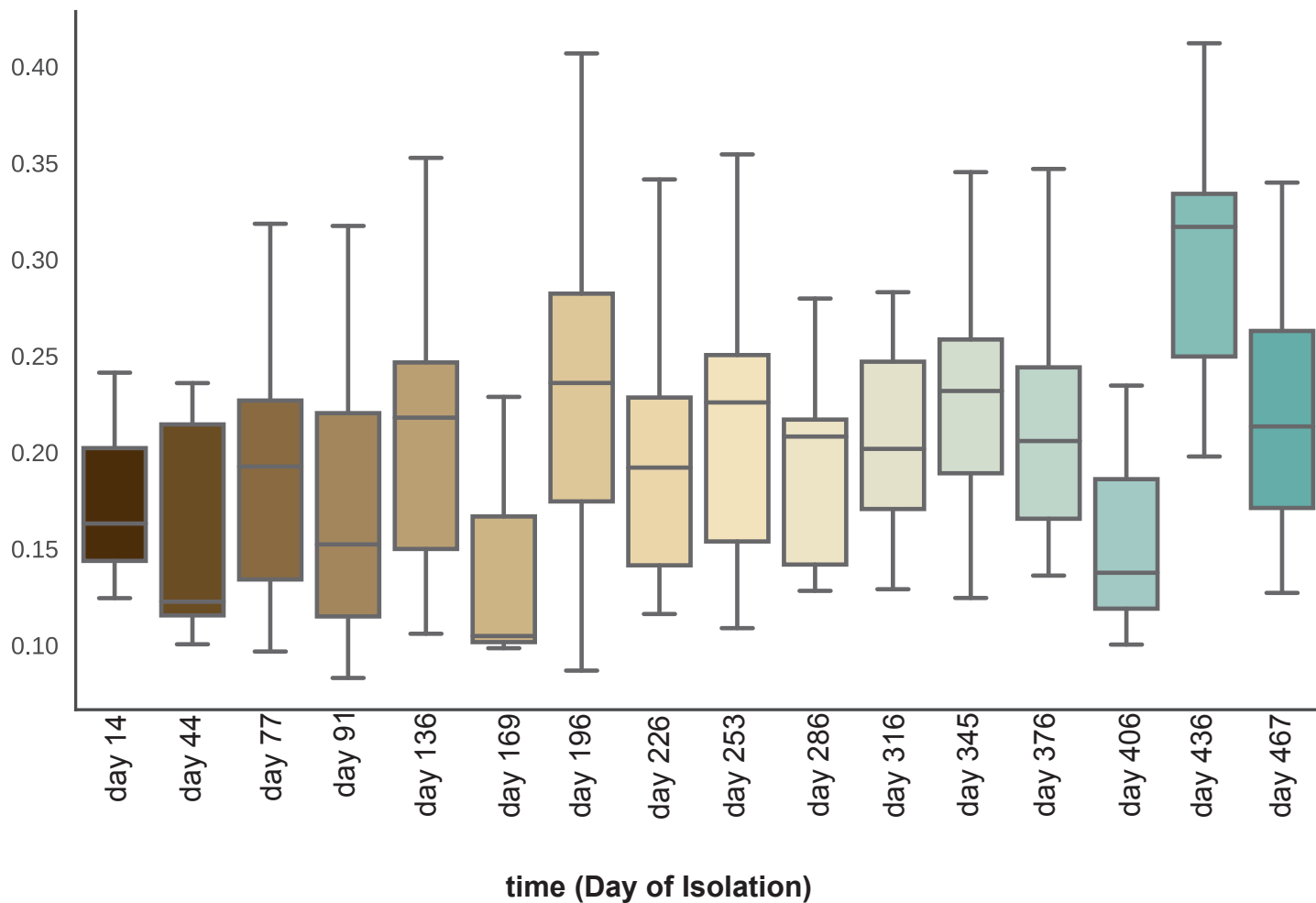

Supplement: Supplementary file 21 — Showing box and whisker plots of weighted unifrac distances of the NGS dataset according to the day of isolation (time). (PDF 169 kb) [file 40168_2017_345_MOESM21_ESM.pdf]

# Pathogens

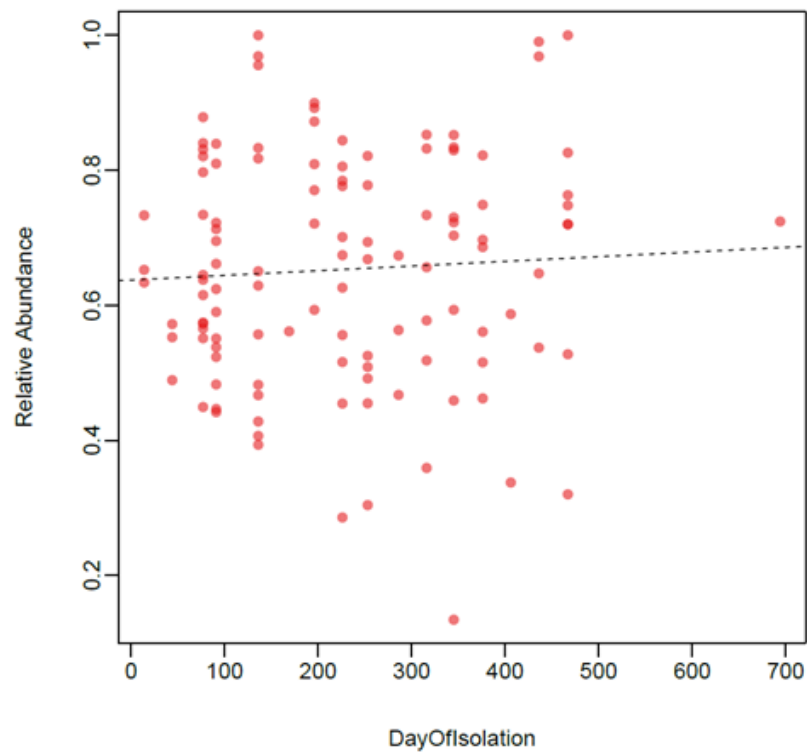

# Stress tolerance

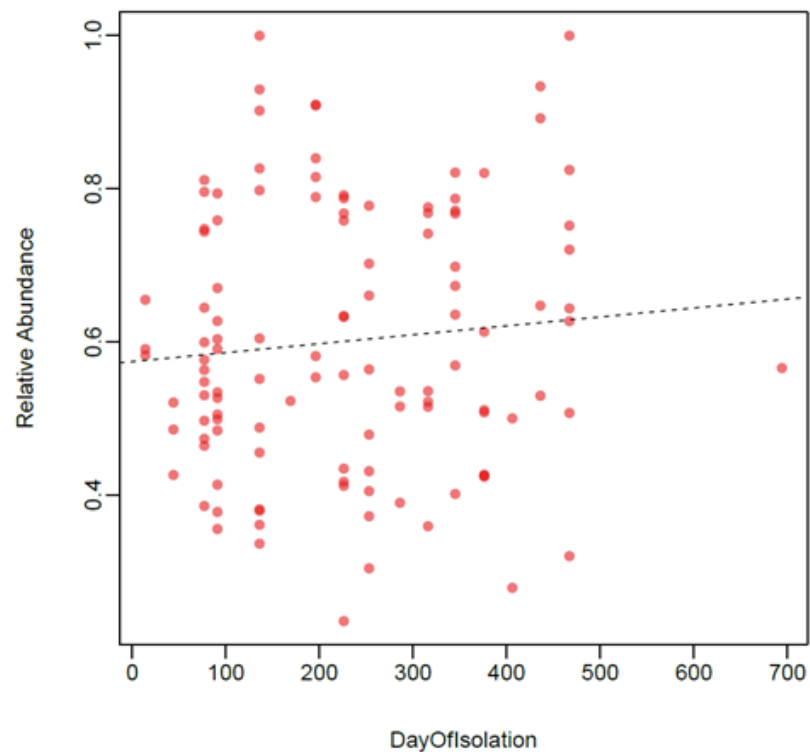

# Mobile Elements

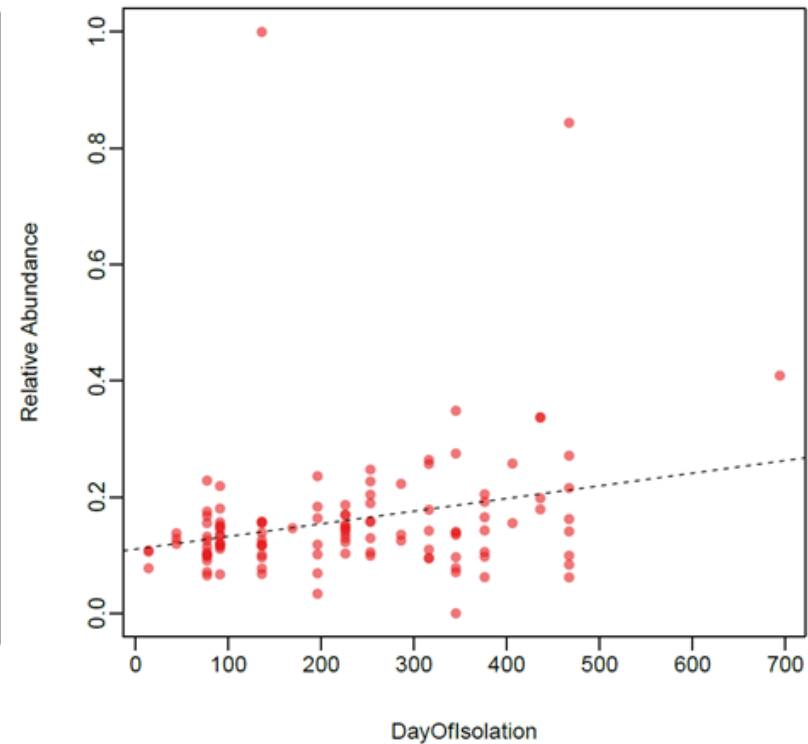

Supplement: Supplementary file 22 — BugBase analyses, based on the NGS dataset. Outcome is grouped according the time-point of sampling (“Day of isolation”, x-axis). The relative abundance is given on the y-axis. “Mobile Elements” refers to bacteria, most probably carrying mobile elements. (PDF 95 kb) [file 40168_2017_345_MOESM22_ESM.pdf]
