# Supplementary material for: Preparing for the crewed Mars journey: microbiota dynamics in the confined Mars500 habitat during simulated Mars flight and landing
Source: Microbiome. 2017 Oct 4;5:129. doi: 10.1186/s40168-017-0345-8 (PMC5627443; doi:10.1186/s40168-017-0345-8)
Supplement: Supplementary file 15 — Showing box and whisker plots of weighted unifrac distances of the NGS dataset according to different positions (surface orientations) of the Mars500 habitat. (PDF 189 kb) [file 40168_2017_345_MOESM15_ESM.pdf]

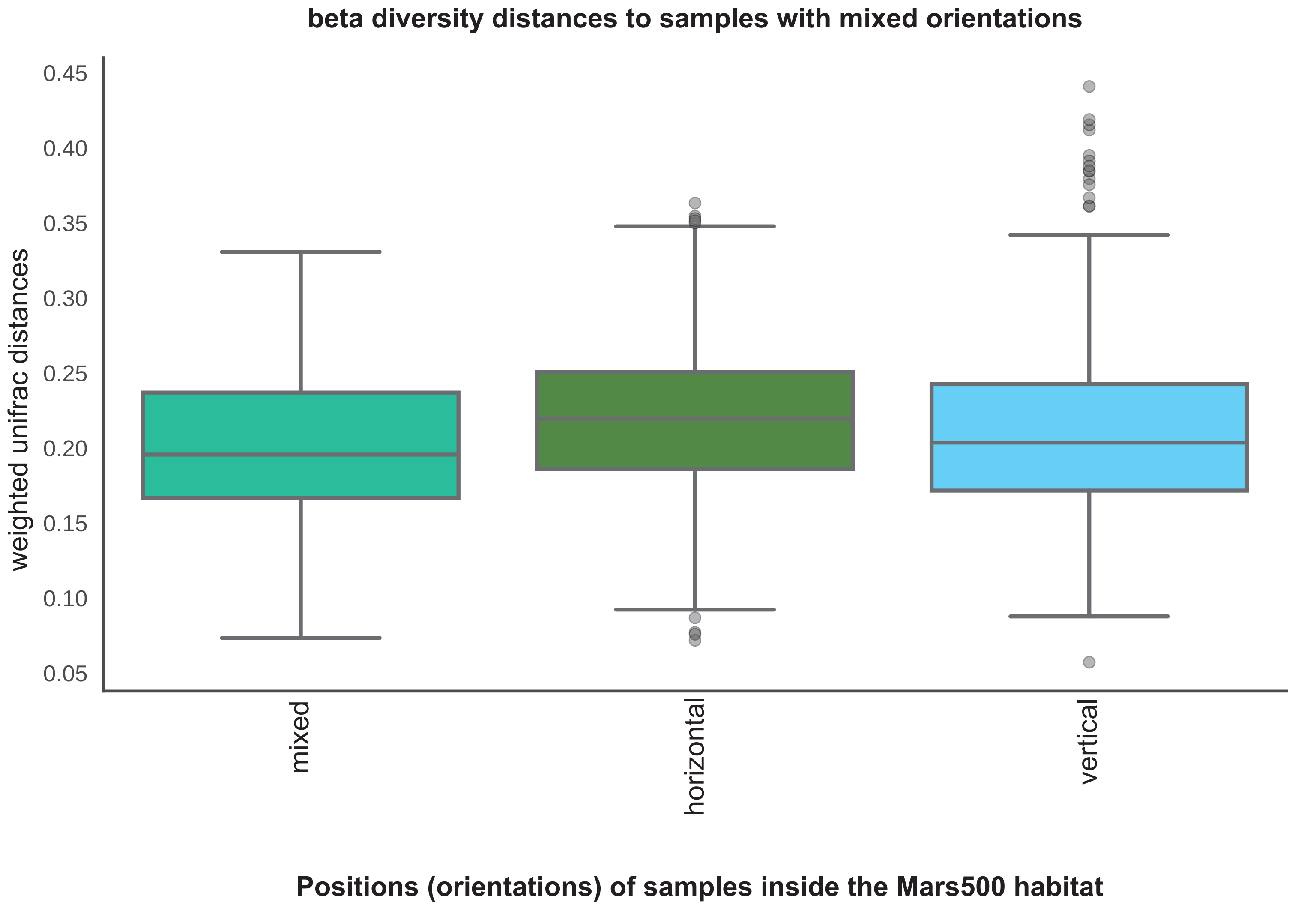

beta diversity distances to samples with mixed orientations
weighted unifrac distances
0.45
0.40
0.35
0.30
0.25
0.20
0.15
0.10
0.05
mixed
horizontal
vertical
Positions (orientations) of samples inside the Mars500 habitat
